# Behavioral Modeling of Human Choices Reveals Dissociable Effects of Physical Effort and Temporal Delay on Reward Devaluation

**DOI:** 10.1371/journal.pcbi.1004116

**Published:** 2015-03-27

**Authors:** Miriam C. Klein-Flügge, Steven W. Kennerley, Ana C. Saraiva, Will D. Penny, Sven Bestmann

**Affiliations:** 1 Sobell Department of Motor Neuroscience and Movement Disorders, UCL Institute of Neurology, University College London (UCL), London, United Kingdom; 2 Wellcome Trust Centre for Neuroimaging, University College London (UCL), London, United Kingdom; 3 Department of Experimental Psychology, University of Oxford, Oxford, United Kingdom; University of Pittsburgh, UNITED STATES

## Abstract

There has been considerable interest from the fields of biology, economics, psychology, and ecology about how decision costs decrease the value of rewarding outcomes. For example, formal descriptions of how reward value changes with increasing temporal delays allow for quantifying individual decision preferences, as in animal species populating different habitats, or normal and clinical human populations. Strikingly, it remains largely unclear how humans evaluate rewards when these are tied to energetic costs, despite the surge of interest in the neural basis of effort-guided decision-making and the prevalence of disorders showing a diminished willingness to exert effort (e.g., depression). One common assumption is that effort discounts reward in a similar way to delay. Here we challenge this assumption by formally comparing competing hypotheses about effort and delay discounting. We used a design specifically optimized to compare discounting behavior for both effort and delay over a wide range of decision costs (Experiment 1). We then additionally characterized the profile of effort discounting free of model assumptions (Experiment 2). Contrary to previous reports, in both experiments effort costs devalued reward in a manner opposite to delay, with small devaluations for lower efforts, and progressively larger devaluations for higher effort-levels (concave shape). Bayesian model comparison confirmed that delay-choices were best predicted by a hyperbolic model, with the largest reward devaluations occurring at shorter delays. In contrast, an altogether different relationship was observed for effort-choices, which were best described by a model of inverse sigmoidal shape that is initially concave. Our results provide a novel characterization of human effort discounting behavior and its first dissociation from delay discounting. This enables accurate modelling of cost-benefit decisions, a prerequisite for the investigation of the neural underpinnings of effort-guided choice and for understanding the deficits in clinical disorders characterized by behavioral inactivity.

## Author Summary

One of the main functions of the brain is to select sequences of actions that lead to rewarding outcomes (e.g., food). However, such rewards are often not readily available; instead physical effort may be required to obtain them, or their arrival may be delayed. The ability to integrate the costs and benefits of potential courses of action is severely impaired in several common disorders, such as depression and schizophrenia. Mathematical models can describe how individuals depreciate rewards based on the costs associated with them. For example, models of how a reward loses appeal with increasing temporal delays can provide individual impulsivity scores, and can serve as a predictor of financial mismanagement. To date, there is no established model to describe accurately how humans depreciate rewards when obtaining them requires physical effort. This is surprising given the prevalence of disorders related to a diminished willingness to exert effort. Here we derive a biologically plausible mathematical model that can describe how healthy humans make decisions tied to physical efforts. We show that effort and delay influence reward valuation in different ways, contrary to common assumptions. Our model will be important for characterizing decision-making deficits in clinical disorders characterized by behavioral inactivity.

## Introduction

Many of the choices that humans and animals make every day depend on cost-benefit analyses. There has been considerable interest from the fields of biology, economics, psychology and behavioral ecology about how decision costs—in particular the delay and uncertainty associated with a rewarding outcome—decrease the subjective value of the expected outcome [1–9]. For example, there is now ample evidence that rewards are hyperbolically discounted by delays [10–12]. This implies that an additional delay in receiving reward has a large effect when added to a short delay, but a small effect when added to a long delay. Similarly, the probability with which reward is received is subjectively distorted such that small probabilities are over-weighted and large probabilities under-weighted [1,13]. Understanding how different decision costs influence human reward discounting has obvious implications for economic decisions, and for clinical disorders, such as anxiety and impulsivity disorders, depression, gambling, and addiction [14–21], in which deficits in cost-benefit decision-making are hallmark features of the disease.

Intriguingly, how physical effort influences our choices has been studied far less than other decision costs, and the underlying discounting effect of effort on behavior remains unclear. This is particularly surprising given the recent interest in the neural and neurochemical mechanisms involved in effort-based choice [22–31], and the fact that a diminished willingness to exert effort is a key signature of several clinical disorders such as apathy, abulia, negative-symptom schizophrenia and depression [32–36]. The most commonly applied models of effort discounting assume that effort costs decrease reward value either linearly [31,37] or hyperbolically [29,38,39], and thus in the same way as delay (although see [40]). However, in the few cases where effort discounting has been examined, a limited number of effort levels were used, contrary to the continuum of effort-based choices that humans and animals experience every day, and the assumption that effort discounting resembles delay discounting has not been formally tested against alternative models.

Indeed, several theoretical arguments suggest that individuals may incorporate effort costs into their decisions in a different way to delay costs. First, the perceived sense of effort increases by a small amount for small efforts, which are easy to execute and energetically inconsequential, but more steeply when efforts become exhausting. More specifically, there is a reduced discriminative ability at lower efforts, and it has been suggested that perceived effort increases as a power function of the required force [41,42]. This would imply an initially concave discounting curve, rather than a hyperbolic function. Second, the willingness to travel further or to wait for rewards differs between animal species [6], providing some indication that the willingness to exert effort or to wait for rewards may not correlate between species or individuals. Finally, from a psychological perspective, it has been argued that the two costs differ fundamentally with regard to what the cost is ascribed to: effort costs are ascribed to actions, whereas delay costs (when not caused by movement times) are ascribed to outcomes [43, but see 44,45]. These dissociable psychological constructs are supported by both neurophysiological and neuropsychological data: lesions of anterior cingulate cortex (ACC)—an area implicated in decision-making and action selection which connects strongly to the motor system—cause deficits in effort-based, but not delay-based, choice behavior, while lesions to the orbitofrontal cortex (OFC)—an area implicated in reward processing but that does not connect with the motor system—cause deficits in delay-based, but not effort-based, decision-making [46].

The hyperbolic effect of delay discounting has been used extensively as a model for self-control and provides a powerful tool to quantify decision-making differences in the normal and clinical populations (e.g., impulsivity, addiction), and even as a predictor of financial mismanagement [47–56]. Establishing a formal model that describes how effort discounts rewards may provide an analogous tool for quantifying behavioral changes in clinical disorders associated with motivational disturbances including reduced physical activity (i.e., apathy, depression, fatigue, abulia)[9], but may also help to understand how reinforcement can influence effortful neurorehabilitation regimes following brain injury. With this aim, we directly compared how effort and delay costs discount the subjective value of rewards. Based on the evidence above, we hypothesized that value discounting of effortful options would be dissociable from that of delayed options. To address this, we directly compared the performance of competing behavioral models on choices involving effort and delay costs (Experiment 1). In a second step, we determined how effort costs affect reward valuation free of any assumptions about the discounting model (Experiment 2). Data from both experiments supported our hypothesis that reward devaluations caused by energetic costs differ from reward devaluations caused by temporal delays, and therefore cannot be described using the same convex hyperbolic model. Instead, we found that effort discounting is best described by a model of inverse sigmoidal shape that is initially concave, rather than convex as previously proposed.

## Results

### Experiment 1: Dissociating effort and delay discounting

**Fig 1:**
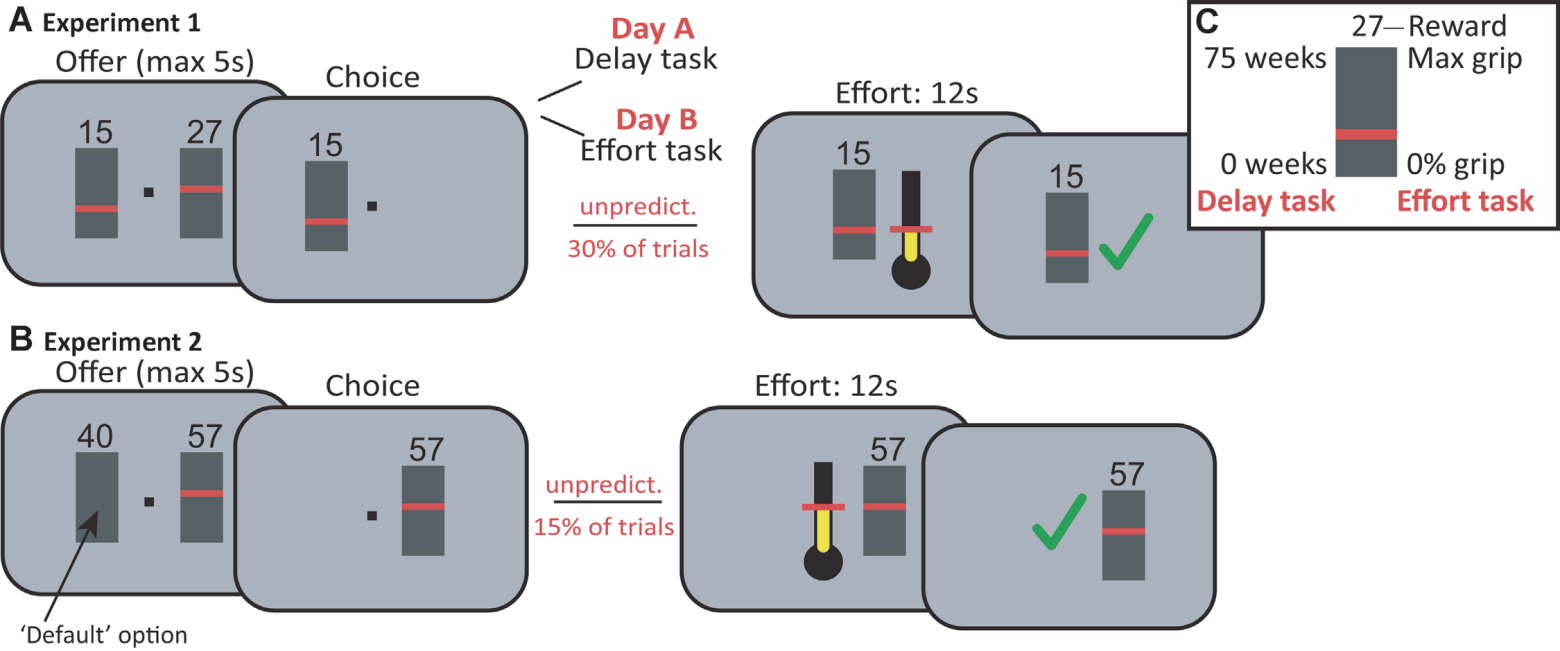
Task: Effort and delay discounting task. **A,** In **Experiment 1**, participants chose between a higher-reward/higher-cost (HRHC) option and a lower-reward/lower-cost option (LRLC; reward magnitude = number; cost level = height of red bar). Magnitudes and costs of both options varied from trial to trial. To directly dissociate effort and delay discounting, decision costs entailed a fixed-duration 12s power grip adjusted to individuals’ maximum grip force on one day (effort task), and a delay to reward (0–75 weeks) on the other day (delay task). Efforts were exerted on an unpredictable 30% of trials (∼15% per hand). **B**, In **Experiment 2**, participants made choices between a ‘default’ option of 40p (block1) or £2 (block2) available for no effort (left) and an alternative option of varying effort and magnitude (right). Participants’ points of subjective indifference were obtained by independently adjusting the magnitude of the alternative option for six levels of effort ([0.15 0.25 0.4 0.55 0.75 1]) across trials, using interleaved staircases. Again, decision costs entailed a 12s grip at the indicated force level. Efforts were exerted using the right hand on an unpredictable 15% of trials, thus avoiding fatigue, and feedback was given upon completion of the required force level for 12 seconds. **C,** The height of the red bar signaled the cost, with the top of the grey box, i.e. the maximum cost, corresponding to 75 weeks (delay task, Exp 1) or a participant’s maximum 12s-grip force. All data figures display costs according to this scale. The reward size was displayed as a number above the cost display. The information in this box was explained to the participant, but was not visible on the screen.

In Experiment 1, we sought to directly dissociate between effort and delay discounting. To this end, choice stimuli were optimized to distinguish between competing models and involved continuous levels of effort. Choices were made between two options associated with varying reward magnitudes and efforts. This required participants to evaluate and compare the subjective values of both offers (Fig. 1A). Physical efforts involved exerting a force grip on a custom-made grip device, and force levels were adjusted to the maximum voluntary contraction (MVC) of each participant. To avoid fatigue, only a subset of randomly chosen trials required participants to exert effort (realized trials), and only the gains accumulated on these trials contributed to participants’ payment. On these trials participants were required to produce a power grip with the force level of the chosen option for a fixed duration of 12s. Thus, effort costs were unconfounded from delay costs. In a separate session, the same participants performed an identical choice task but with rewards involving delay (0–75 weeks), unconfounded by physical effort. A single pseudo-randomly chosen trial was paid out in the delay task, whereas money was accumulated from all grip trials during the effort task, as explained above. Overall, participants (n = 23) chose the higher effort option on 54 ± 5% (mean ± SEM) of trials, and the more delayed option on 52 ± 3% of trials. This demonstrates that effort and delay were both factored into the choices, and to a similar extent. A logistic regression of participant’s choices furthermore showed that costs and magnitudes both influenced choices (S1B Fig.). Moreover, the pattern of choice response times reflected an integration of costs and benefits (S1 Text and S1 Fig.).

**Fig 2:**
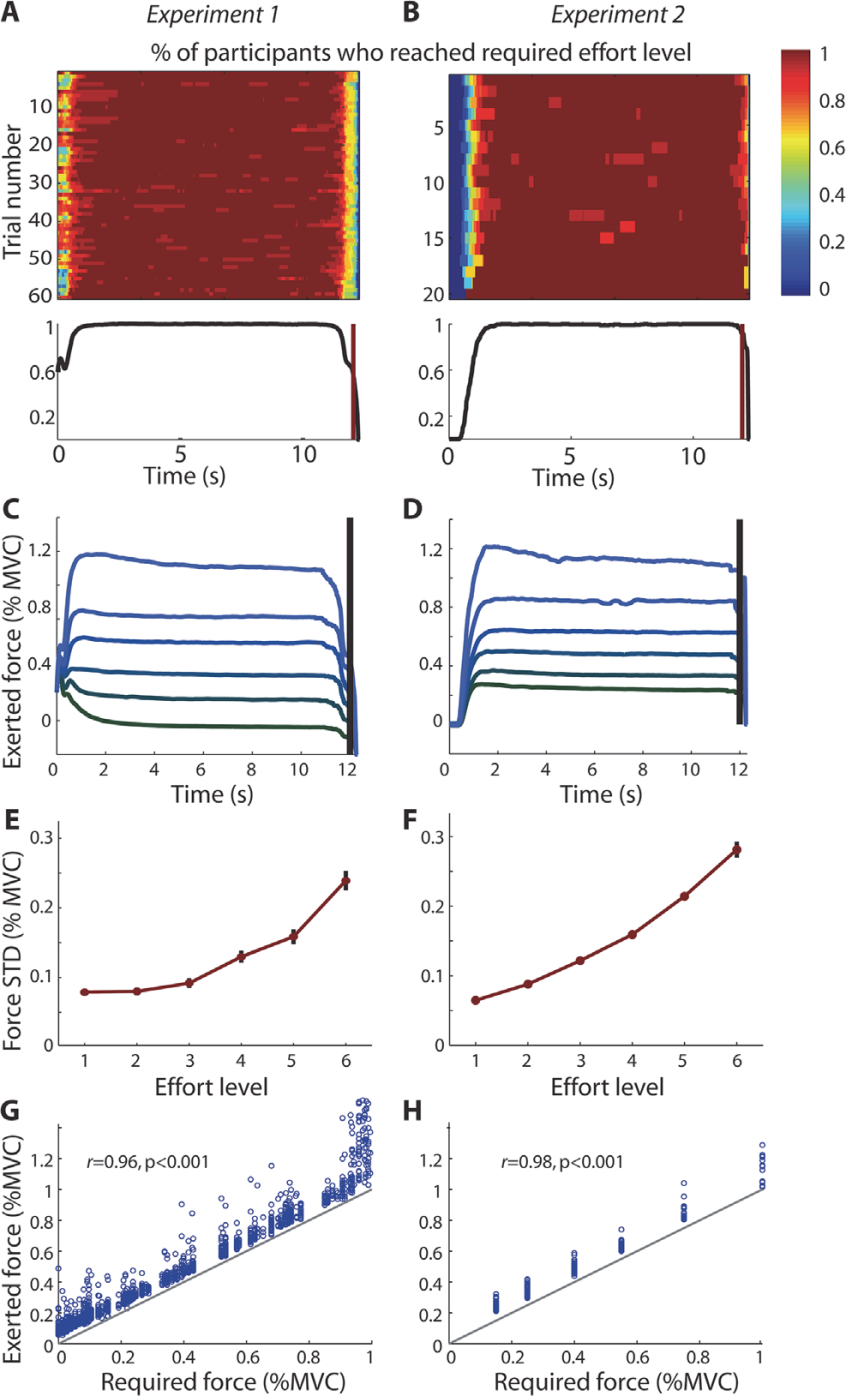
Force-grips: Completion of force-grip on effort trials. Force traces from trials in which a 12s grip was exerted in Experiment 2 (A,C,E,G) and Experiment 1 (B,D,F,H). **(A,B)** Trial-by-trial (top) and trial-average (bottom) percentage of participants that had reached the required effort level (color bar: 0 = no participant, 1 = all participants). **(C,D)** Group average 12s force trace for six different effort levels, expressed as percentage of participants’ maximum voluntary contraction (MVC; Experiment 1: equally sized bins between [0,1]; Experiment 2: [0.15 0.25 0.4 0.55 0.75 1];). There was an offset at t = 0 in Experiment 1 because the 12s-effort directly followed the response which was indicated by briefly squeezing the gripper of the corresponding hand at a level of 0.35 (see Materials and Methods). **(E,F)** Within-trace average variability (STD = standard deviation) for the same six effort levels increases with increasing difficulty (group average ± SEM). **(G,H)** Required force levels are plotted against mean exerted force levels for every effort trial, showing a strong correlation in both experiments. The produced force consistently exceeded the required force level by a small amount because participants were instructed to maintain their force just above the target bar and because they kept a ‘safety margin’ to account for slight variations in force.

Participants were successful in producing the 12s-grip on 97.32±1.2% of realized effort trials (criterion: maintaining the grip force indicated by the bar-height for at least 80% of the 12s duration; Fig. 2A, C). This result shows that required force levels had been adequately adjusted to each individual’s maximum grip force. The average reaction time (RT) to reach the required force level was 727 ±105ms, determined here as the time spent above the required force level, but exceeding it by at most 20% (see Materials and Methods). The average time spent above the required level was 94.95±0.5% (11.39s of 12s). The variability of the produced force output scaled with the force level (six equally sized bins of effort levels: F(5,85) = 112.61, p<0.001, ɳ_p_^2^ = 0.87; Fig. 2E), the produced forces correlated with required force levels (r = 0.96, p<0.001; Fig. 2G), and fatigue did not affect choice behavior (see below).

**Fig 3:**
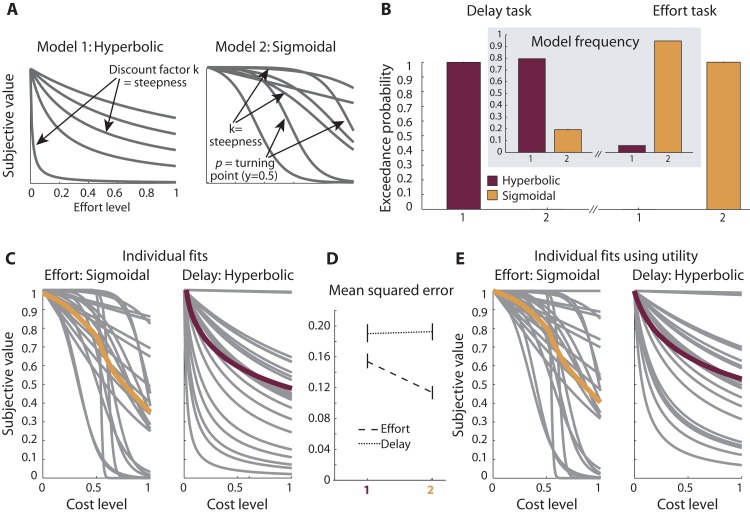
Experiment 1: Comparison of effort and delay discounting. **A**, Two models were fitted to participants’ choice data; the hyperbolic model has previously been proposed to characterize effort and delay discounting. The sigmoidal model was included because it fulfills two particular features: it can obtain initially concave shapes, in line with work showing that the sense of effort increases as a power function of the target force with decreasing sensitivity at lower effort levels; and it entails a turning point after which effort discounting becomes progressively less steep. The equations are as follows: hyperbolic: V = M/(1+kC), sigmoidal: V = M (1- (1/(1+exp(-k*(C-p)))- 1/(1+exp(k*p))) (1 + 1/exp(k*p))). **B**, Bayesian model comparison of the hyperbolic and the sigmoidal model showed a clear dissociation: the hyperbolic model best explained delay-based choices (left), whereas the sigmoidal model best explained effort-based choices (right). **C**, Individual and average fits of the sigmoidal winning model for effort and the hyperbolic winning model for delay. **D**, Mean squared error, indicating the goodness of fit of the two competing models for the effort and delay task. **E**, Individual and average model fits, as in **C**, but these fits were obtained by using the utility instead of reward magnitude during parameter estimation. This has a negligible effect on the shape of discounting we observe.

Our main aim was to directly compare choice behavior when rewards were tied to two different types of decision costs, physical effort versus temporal delay. To this end, we initially compared two behavioral models of subjective discounted value, and contrasted their performance for choices on the effort and delay task. The first was the hyperbolic model widely accepted as the best characterization of delay discounting behavior [8,10–12], but which has also been suggested for effort discounting [29] (Fig. 3A). The second was a sigmoidal model with an initially concave shape and a flexible turning point (see Materials and Methods). Such a model can accommodate discounting behaviour in which effort increases at low effort levels have a smaller effect on value than increases at higher effort levels. Such a behaviour would be consistent with studies showing that the perceived sense of effort increases as a power function of the exerted force level, with a reduced sensitivity to lower compared to higher efforts [41,42]. To strengthen our claim that effort discounting is concave and dissociable from delay discounting, we also performed a comparison of a larger set of models. This comparison included three one-parameter models previously suggested for effort discounting (hyperbolic [29]; linear [37]; quadratic [40]), and a two-parameter power function, with the latter two sharing the initially concave nature with the inverse sigmoidal model (see S2A–B Fig.).

**Fig 4:**
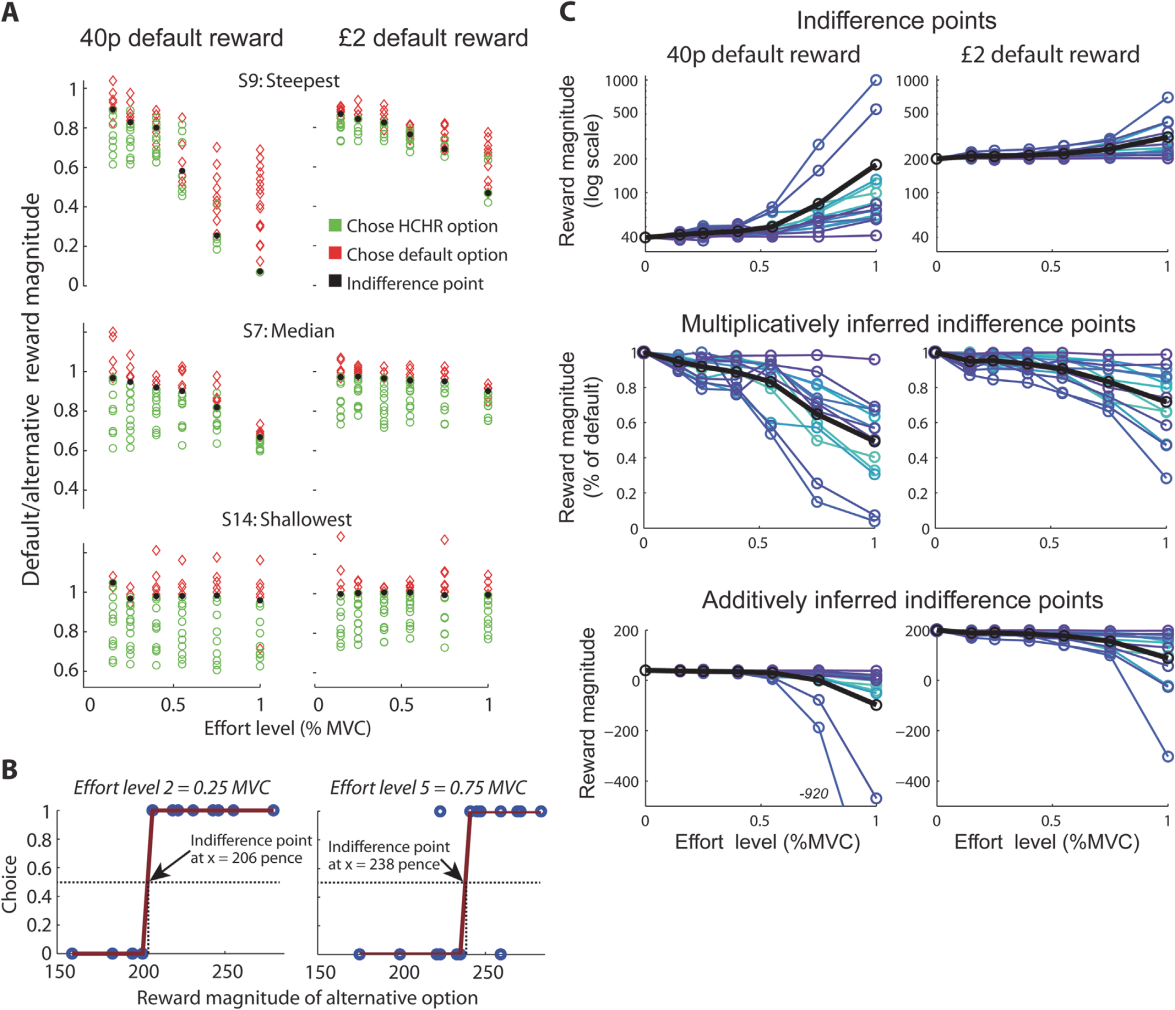
Experiment 2: Model-free analysis of effort discounting. **A**, Offered stimuli and choices from Experiment 2 are shown for the participants with the steepest, median and shallowest discounting, respectively, separately for the block with a default reward magnitude of 40p (left) and £2 (right). Stimuli were dynamically adjusted using a hidden staircase procedure to identify individual participant’s indifference points. For each effort level, the offered reward magnitude is displayed as the default option divided by the alternative option, thus yielding values <1 when the alternative option was associated with more reward (for alternative representations of indifference points, see **panel C**). Choices of the higher-reward/higher-cost option are indicated in green, choices of the effortless default option in red, and inferred multiplicative indifference points in black. **B,** Procedure for determining indifference points: A sigmoid function was fitted separately to the choices generated at each effort level (y axis: 0 = default option chosen, 1 = alternative option chosen), given the reward magnitude of the alternative option (x axis). The point of subjective indifference between the default and the alternative option was defined as the reward magnitude at which where the sigmoid crossed y = 0.5. Exemplary choices and fits are shown for one subject and two effort levels in the £2 (200p) block. **C**, Individual (coloured) and average (black) group indifference points show that effort discounting follows a concave, rather than convex, shape and scales with offer magnitude (40p vs. £2). Shown are the raw untransformed indifference magnitudes (top), a multiplicative representation of indifference points, i.e. default magnitude / indifferent magnitude (middle), and an additive representation of indifference points, i.e. (default magnitude—(indifferent magnitude—default magnitude)) (bottom). A multiplicative representation yields a percentage decrease, whereas an additive representation reflects the absolute decrease in value, which can easily lead to negative values. These data do not depend on any model fits and in all cases clearly reflect the concave nature of effort discounting, the main message of this study.

For each model, participants’ choices were fitted using a Bayesian estimation procedure. Comparison between the resulting fits was conducted using Bayesian model comparison (see Materials and Methods). The resulting exceedance probability (xp) indicates the likelihood of each model to be the most frequently occurring model in the population, and the mean of the posterior distribution (mp) provides an estimate of the frequency with which the model appears in the population. As expected, the hyperbolic model best explained participants’ choice behavior in the delay task (xp = 0.99, mp = 0.80; Fig. 3B). By contrast, the sigmoidal model outperformed the hyperbolic model in the effort task (xp = 1, mp = 0.96; Fig. 3B). We note that our choice set was optimized to distinguish hyperbolic from quadratic/concave shapes (see Materials and Methods). Therefore the quadratic model suggested previously [40] and which is also concave, also provided a good explanation of effort-based choices, and explained our data substantially better than the hyperbolic model (S2 Fig.). The same held true for a two-parameter power function with flexible curvature and exponent (see S2 Fig., C): it performed almost equally well as the sigmoidal model (for a comparison of all five models, see S2 Fig., B). Crucially, all three concave models (quadratic, two-parameter power, sigmoidal) clearly outperformed the hyperbolic model (all xp>0.99, mp>0.85). This reinforces our main result that effort discounting is best characterized by concave, rather than convex, models.

Note that the quadratic and two-parameter power functions do not asymptote when efforts become increasingly high. By contrast, the sigmoidal model has an asymptote which may be a critical difference between these models. We argue that it is a plausible feature for an effort discounting model to have an asymptote because very high effort levels that cannot be reached are likely to become non-discriminable and have a similar impact on value. However, we note that this precise feature of the model remains to be tested more systematically in future studies, especially with regards to the values it converges to.

Formally, the quadratic and two-parameter power functions are additive models. This means that their native definition can be reduced to the form V = M+f(C). By contrast, the sigmoidal model proposed here is a multiplicative model and of the form V = M*f(C). Forthcoming investigations will need to determine whether, or under which conditions, effort discounting is of additive or multiplicative nature. For example, using a fixed value for the effortful option and a variable value for the non-effortful option would enable one to distinguish between the two alternatives.

Because the sigmoidal model can also describe shapes that have an early turning point (and therefore take a concave shape only for the initial small part of the curve, but are otherwise convex within the range between [0,1]), we directly compared the fits obtained from this model for both the delay and effort task. This revealed that the same model generated opposite discounting shapes: the majority of participants had predominantly convex shapes for delay discounting (turning point < 0.3 in 17 out of 23 participants), but initially concave shapes for effort discounting (turning point > 0.5 in 17 out of 23 participants; Figs. 3C and S2D).

The percentage of choices correctly predicted by the two models were 84.0 ± 1.3% for the winning sigmoidal model (vs. 76.6 ± 1.6% for the hyperbolic model) for the effort task, and 71.0 ± 1.8% for the winning hyperbolic model (vs. 70.9 ± 1.9% for the sigmoidal model) for delay, respectively (S3A Fig.; Table 1). The sigmoidal model explained a larger percentage of choices, compared to the hyperbolic model, in 18 of 23 participants for the effort task, and the hyperbolic model in 6 of 23 participants for the delay task (at least equally many choices: 22 vs 12 of 23, respectively; Table 1). The percentage of choices predicted by both models is shown in S3 Fig. The sigmoidal model predicted significantly more choices than the hyperbolic model for effort-choices (t(22) = 4.69, p<0.001, Cohen’s d = 1.17), but the two models did not differ for delay-choices (p = 0.8). This is importantly not our measure for determining the winning model. Only the formal model comparison presented above took into account additional model parameters. Fig. 3C shows the individual and average fits of the winning models for both tasks. The average model parameters were k = 10.49±4.28 and *p* = 0.70±0.09 for the sigmoidal model for effort, and k = 4.86±2.20 for the hyperbolic model for delay (see S1 Table). Overall, Experiment 1 confirmed that effort discounting is best described using an initially concave curve in cases when both options involve efforts, and it established that humans assign subjective value to reward differently when choices involve effort versus delay costs.

**Table 1:** Choices explained by the model (Experiment 1).

% choices correctly predicted: Effort and delay: Experiment 1				
	EFFORT TASK		DELAY TASK	
	hyperbolic	sigmoidal	hyperbolic	sigmoidal
**s1**	0.67	0.92	0.81	0.79
**s2**	0.73	0.77	0.83	0.84
**s3**	0.70	0.76	0.65	0.65
**s4**	0.61	0.92	0.69	0.69
**s5**	0.69	0.87	0.66	0.67
**s6**	0.78	0.80	0.71	0.71
**s7**	0.82	0.80	0.73	0.72
**s8**	0.92	0.93	0.65	0.65
**s9**	0.73	0.86	0.87	0.88
**s10**	0.91	0.91	0.87	0.79
**s11**	0.77	0.85	0.86	0.88
**s12**	0.73	0.85	0.66	0.67
**s13**	0.68	0.80	0.67	0.67
**s14**	0.72	0.74	0.64	0.65
**s15**	0.86	0.88	0.70	0.72
**s16**	0.79	0.91	0.63	0.63
**s17**	0.80	0.94	0.86	0.87
**s18**	0.76	0.79	0.70	0.68
**s19**	0.73	0.73	0.65	0.66
**s20**	0.76	0.76	0.65	0.68
**s21**	0.77	0.84	0.61	0.59
**s22**	0.85	0.85	0.64	0.60
**s23**	0.87	0.87	0.63	0.63
**Mean**	0.77	0.84	0.71	0.71
**SEM**	0.02	0.01	0.02	0.02

For each subject, the table shows the percentage of choices in Experiment 1 that were correctly predicted by the hyperbolic and sigmoidal model (compare Figs. 3 and S3A), for both the effort (left) and delay (right) task.

To examine whether individuals’ discounting behavior was similar across the two types of costs, we tested for a correlation of the estimated model-parameters between the two tasks. We performed this test for each of the parameters of the respective winning models, but also included comparisons of the same model (hyperbolic, sigmoid) applied to both cost types. None of these tests showed any evidence for a relationship between effort and delay discounting (all *p*>0.23, abs(*r*)<0.26), indicating that participants did not exhibit similar (or opposing) tendencies to discount reward when it was associated with effort versus delay costs. The full table of p-values and correlation coefficients is reported in Table 2.

**Table 2:** No evidence for a relationship between effort and delay discounting.

			P-values			Correlation coefficients		
			Effort			Effort		
			hyperbolic	sigmoidal		hyperbolic	sigmoidal	
			*k*	*k*	*p*	*k*	*k*	*p*
Delay	Hyperbolic	*k*	0.60	0.56	0.95	-0.11	-0.13	0.02
	Sigmoidal	*k*		0.32	0.44		-0.22	0.17
		*p*		0.50	0.45		-0.15	0.17

P-values and correlation coefficients of the correlation between model parameters (from Table 1 in S1 Text; Experiment 1; *k* is hyperbolic discount rate; *k* and *p* are sigmoidal steepness and turning point) show no evidence for a relationship between individuals’ discounting tendencies for effort and delay costs.

### Using utility instead of magnitude

It is possible that effort discounting operates in units of utility, rather than units of reward magnitude (here pounds and pence). The utility function is typically concave for the average human participant [57,58]. Therefore, to ensure that the shape of effort discounting would be unchanged when using utility instead of raw magnitude, we repeated the model fits using a generic utility parameter (see Materials and Methods). This revealed no qualitative change in the shape of the discount curve (Fig. 3E). Similarly, when all models were fitted using utility instead of magnitude, this did not change the conclusions from the model comparisons reported above (xp = 1, mp = 0.96 for sigmoidal model, compared to hyperbolic, for effort, and xp = 0.99, mp = 0.81 for the hyperbolic model, compared to sigmoidal, for delay as in Fig. 3B).

### Results are not trivially explained by a larger number of model parameters, the exerted force, or fatigue

Our Bayesian Model Comparison accounts for model complexity by using the Kullback-Leibler divergence between prior and posterior densities over parameters. These divergences tend to be larger as the number of parameters increases, thus automatically penalizing larger models. But, for example, if the marginal distribution over one of the model parameters does not change as a result of model fitting, a penalty will not be paid for it. To clarify this issue, we decomposed the log model evidences into accuracy and complexity terms so as to be clear about what is driving each of the model comparisons (Materials and Methods; see **S2 Table**). This showed that the additional complexity of the sigmoidal model was more than compensated for by an increase in accuracy. By contrast, for delay the hyperbolic model had a higher accuracy than the sigmoidal model, despite having one less parameter.

The force exerted during effort production trials slightly exceeded the required force and became more variable with increasing effort levels (Fig. 2, G-H). We conducted a control analysis to test whether this non-linear variability increase in the produced force could explain the concave shape of discounting observed for effort costs. Bayesian parameter estimation and model comparison were performed as before, but using the force level produced on a given trial instead of the required target force. Because many trials did not require an effort production, we fitted a quadratic model to the force measured in all effort production trials for every participant (Fig. 2, G-H). This subject-specific fit was used to predict the ‘produced force’ for each trial. None of our results changed as a result of using the (predicted) produced, rather than required, force level: as before, model comparison showed that the sigmoidal model best accounted for the data, and the resulting shape of discounting was still concave (see S3 Table; S4 Fig.). Thus, effort discounting is concave independent of whether participant’s evaluation relies on the required or the predicted produced effort cost.

We furthermore assessed whether our results could have been influenced by fatigue. To this end, we distinguished two types of fatigue: trial-by-trial fatigue, which may reduce choices of the higher-reward/higher-cost (HRHC) option in trials immediately following a 12s-grip (or potentially only a hard 12s grip), and accumulating fatigue, which may lead to gradually decreasing HRHC choices as the experiment progresses. The percentage of HRHC choices did not differ on post-effort versus post-no-effort trials (p = 0.84; see S4 Table). Trials following hard as opposed to easy effort trials also did not differ in the percentage of HRHC choices (median split separates trials at a force of 0.35% MVC: n_easy_ = 30.6±2.4, n_hard_ = 27.7±2.4; p = 0.95). Finally, the percentage of HRHC options did not differ between the first and second half of the experiment (53±5% and 56±5% HRHC choices; p = 0.23), indicating that participants did not become more effort-averse as the experiment progressed.

### Interpreting the percentage of explained choices

The percentage of choices correctly predicted by the winning sigmoidal model differed by about 7% from those predicted by the hyperbolic model, indicating that a majority of choices could be explained by both models. Critically, the magnitude of such a difference in explanatory power cannot be taken as a direct measure of how much better a model can, in general, explain certain types of choices. Whether a choice can be better predicted by one model compared to another not only depends on the model, but also on the choice in question.

To illustrate this point, we compared the percentage of choices correctly predicted by the hyperbolic and sigmoidal model on a subset of our choice stimuli, namely 36 choices where both options were in the lower cost range or both options were in the higher range of cost levels (S5B Fig.). These choices should be particularly good at distinguishing between the two models. Indeed, while the percentage of predicted choices on the entire stimulus set was 84.0 ± 1.3% vs. 76.6 ± 1.6% (for effort) for the sigmoidal and hyperbolic model (difference of 7.4%), the difference in predictive power was >12% on this subset of choices, specifically 81.9 ± 2.0% versus 69.4 ± 2.6%, respectively.

A strength of the present study was that choice stimuli were kept constant for two tasks and all participants, but as a consequence, the stimuli did not maximally distinguish between competing models in every participant. We note that the differences in predictive performance of the present models are nevertheless comparable, or exceed that of established models (e.g., hyperbolic versus exponential model for delay discounting [59]; an improved volatility learning model over a standard reinforcement learning model; see [60]). Collectively, this demonstrates that the sigmoidal model is superior to the hyperbolic model because it consistently predicts more choices across subjects.

Another observation was that participants were less consistent in their choices in the delay task, and therefore both models compared here overall predicted fewer choices correctly in this task, compared to the effort task. One of the reasons for this difference in consistency could be that our choice display which used the height of a bar to present decision costs was more appropriate for representing efforts than for representing temporal delays. To test this hypothesis, a subset of participants (14 out of 23) completed the delay task again, using identical stimuli, except that delays were displayed using words (‘6 weeks’) instead of horizontal bars. This drastically increased the percentage of correctly predicted choices. S3B Fig. shows the percentage of predicted choices for this subset of participants in the original and modified task versions (delay as bar: 71.9 ± 2.3%; delay in words: 85.5 ± 1.9%). Importantly, in either task version, the hyperbolic model outperformed the sigmoidal model (Bayesian model comparison for delay task involving words: xp = 0.98; mp = 0.75), consistent with a large literature on delay-based choices.

### Experiment 2: Indifference curves for effort discounting

Experiment 2 aimed at establishing how rewards are devalued when requiring physical effort using a model-free approach. Participants made choices between an option for which reward magnitude and effort level varied from trial to trial, and a ‘default’ option with constant reward magnitude that never required effort exertion (block1: 40 pence, block2: £2; Fig. 1B). The advantage of keeping one option constant was that stimuli only varied along two dimensions (one effort and one reward magnitude). As a result, choice preferences could be easily visualized (Fig. 4A,C) and indifference points determined, free of any assumptions about the precise discounting function. This allowed us to validate our main conclusion from Experiment 1 that effort discounting is initially concave. Physical efforts again involved exerting a 12s-force grip adjusted to the MVC of each participant. For each of the six levels of effort, the reward magnitude of the variable option was adjusted on a trial-by-trial basis, based on the participants’ choices, using a hidden staircase procedure (median magnitude range of the variable option: block1: 25±3 to 81±38 pence; block2: 187±5 to 293±23 pence). The shape of effort-discounting was inferred from the point of subjective indifference at which participants assigned equal value to the option involving effort and the default option (see Materials and Methods). This procedure was free of any assumptions about the shape of discounting. We also determined how effort-discounting scales with different levels of reward magnitude by comparing discounting behavior between the 40p and £2 block.

As in Experiment 1, only a subset of randomly chosen trials required participants to exert effort, and only the gains accumulated on these trials contributed to participants’ payment. On these trials participants were required to produce a 12s power grip with the force level of the chosen option. Alternatively, when participants had chosen the default option, they had to wait for 12s without producing any force. This ensured that effort costs were unconfounded from delay costs. On average, 15.8 trials (8.2%) required effort production and participants (n = 14) successfully reached the criterion (pressing the grip-device with at least the force indicated by the bar-height for at least 80% of the time) on 97.2±1.1% of these effort-trials (Fig. 2B, D). This shows that the required force levels had been adequately adjusted to each individual’s maximum grip force. The average RT to reach the required force level was 793±53ms, and the average time spent above the required level was 91±0.4% (10.92 of 12s). The variability of the produced force output increased with increasing force levels (main effect of force level: F(5,25) = 168.86, p<0.001, ɳ_p_^2^ = 0.97; Fig. 2F), and exerted and required force levels were highly correlated (r = 0.98, p<0.001; Fig. 2H). Overall, fatigue did not affect choice behavior (see below).

Fig. 4A shows the choices from three participants with the shallowest, median, and steepest discounting respectively, separately for each of the six levels of effort, and for the 40p and £2 blocks (see Fig. 4B and Materials and Methods for calculation of indifference points). Here the reward magnitude of the varying option is depicted as percentage of the default option, and thus in a multiplicative fashion. The resulting points of subjective indifference are therefore a measure of *relative* subjective value. An alternative way to depict these choices is in an additive fashion, by plotting the difference between the default and varying reward magnitude (see Fig. 4C). Multiplicative and additive representations, as well as the untransformed indifference points for all participants are shown in Fig. 4C. As explained above for Experiment 1, it is yet unclear whether effort discounting is multiplicative or additive in nature. Therefore, either transformation could be valid. But importantly our main conclusion of initially concave effort discounting does not depend on the type of transformation. For the sake of clarity, the next paragraph focuses on the multiplicatively inferred subjective values.

Comparison of the multiplicatively inferred indifference points across effort levels revealed that the majority of participants (10 out of 14) exhibited concave (or ‘parabolic’, see [40]) discounting, which is characterized by initially small decreases in value followed by steeper reward devaluations at higher effort levels; a curve was classified as concave when at least three out of five consecutive indifference points lay above the line connecting (0,1) with the sixth indifference point (Fig. 4C; 3.64±0.20 of 5 inferred indifference points lay above this line for multiplicatively inferred subjective values). On average, this analysis revealed a concave shape for effort discounting (Fig. 4C) both for the lower and higher default reward magnitudes. However, the discounting rate scaled with the absolute level of reward (‘magnitude effect’). Effort discounting was shallower when more reward was at stake (ANOVA of multiplicatively inferred indifference points for default magnitude (2) x effort level (6): all main effects and interactions p<0.001: default magnitude: F(1,13) = 38.35, ɳ_p_^2^ = 0.75; effort level: F(5,65) = 37.12, ɳ_p_^2^ = 0.74; magnitude x effort level: F(5,65) = 19.13, ɳ_p_^2^ = 0.60; Fig. 4C). This shows that effort discounting depends on the offer value, a well-known effect for delay discounting [59,61–64]. Using alternative representations of indifference points (raw or additive) the above conclusions remained the same: the change in value was smallest at lower effort levels, implying an initially concave discount curve, and discounting was shallower for a larger default reward magnitude.

Because there were overall fewer effort trials, fatigue should have contributed less to choices in Experiment 2 compared to Experiment 1. Indeed, choice behavior was not affected by trial-by-trial fatigue. The percentage of HRHC choices did not differ between post-effort trials (i.e., the trials immediately following a trial with realized effort production of 12s), and trials following no-effort trials, matched in magnitude and effort difference (100,000 permutations of random subsets of the same number of trials; p = 0.10). If anything, there were descriptively more HRHC choices following effort trials (64±3% versus 58±2%), suggesting fatigue did not bias choices in a systematic way. Similarly, there was no difference in the percentage of HRHC choices on trials following an easy compared to a hard realized effort (median split; p = 0.69); these trials did not differ in their average magnitude or effort difference.

The experimental design aimed to minimize effects of accumulating fatigue, by realizing efforts on a subset of unpredictable trials only. Moreover, ITIs following realized effort trials were longer and thus minimized carry-over between trials. When comparing the first (block 1) and the second (block 2) half of the experiment, participants actually became more likely to choose HRHC options, but this is trivially explained by the change in the default reward magnitude from 40p to 2£ (t(13) = 3.02, p = 0.01, Cohen’s d = 1.68; Fig. 4C). This outcome also is not compatible with accumulating fatigue.

## Discussion

In the present study we demonstrate that the way in which humans integrate physical effort into the valuation of rewards for making choices differs profoundly from the way they integrate delays to reward. While in relative terms, smaller temporal delays have the largest effect on reward devaluation, low physical effort has a small impact on reward devaluation.

### Effort discounting is concave and differs from delay discounting

First, humans seem to be able to easily tolerate a range of proportionally low energetic costs. In other words, physical efforts perceived as requiring small amounts of energy (e.g., taking the escalator or walking up a small number of stairs) will hardly decrease the value of reward. Only once these costs exceed a level that is subjectively perceived as ‘tiring’ or ‘hard’ (e.g., walking up several flights of stairs) do individuals start to discount rewards more noticeably. Thus, the impact of effort on reward valuation should become stronger with increasing effort levels, consistent with the initially concave discounting shape observed in the majority of our participants. This choice behavior is the opposite of delay discounting behavior, where additional delays have the strongest effect when added to small, compared to large, delays.

More generally, data from two tasks in which effort costs were unconfounded from delay costs provide robust evidence against the notion that effort discounting is best explained by a hyperbolic function as previously suggested [29,38,39]. By using a robust Bayesian modeling approach (Experiment 1), and by directly measuring participants’ indifference points for different effort levels (Experiment 2), our results instead indicate that effort discounting is best characterized by a sigmoidal two-parameter model that allows initially concave discounting shapes. Other work has suggested or implicitly used a linear model of effort discounting [31,37], and, more recently, a quadratic function [40]. It is notable that a close look at some of the published data suggests a discount function of the concave shape proposed here (e.g., compare Fig. 2 in [31], and Fig. 1 in [65]). Critically, we note that previous studies did not directly compare the performance of the hyperbolic or linear model to any alternative models, and did not dissociate choices involving delay and effort costs. Therefore, the question as to whether effort and delay are discounted in similar ways remained unresolved.

The sigmoidal model proposed here provides a superior fit to effort-based choices compared to any of the other models, but there are also several theoretical arguments that justify its choice. A discount function that is concave for achievable effort levels is consistent with work showing that the subjective ‘sense of effort’ increases as a power function of the exerted force level [41,42], and it may directly relate to the underlying physiology. For example the rate coding of muscle units changes little at low force levels, but increases more with increasing effort levels [66,67]. More work is required to test for such relationships and to determine which aspects of effort discounting relate to physiology or inter-individual personality differences. We found that simpler functions fulfilling the concave property of our model, such as a quadratic function with only one parameter, also provide good fits to effort-based choices [40]. Importantly, the concave nature of effort discounting cannot be accounted for by the concave shape of the utility function. When using utility instead of reward magnitude for fitting the model, the concave shape of the effort discounting function is preserved.

The second property of our model is that it entails a turning point after which discounting becomes progressively less steep. When we represent our data multiplicatively (Fig. 4), there is evidence that about half the participants experience such a turning point before 100% MVC (and others may do at higher levels exceeding 100% MVC not tested here), but it is important to note that our task was explicitly designed to distinguish concave from convex shapes. With the benefit of hindsight a design with additional power to determine the tail of the discounting function with confidence would have allowed us to address this issue. Nevertheless, the sigmoidal model outperforms a two-parameter power function with similar flexibility in the lower range of efforts, which suggests that the turning point may be a critical feature. Here we intentionally stayed in the range of efforts a participant can produce with 100% certainty to avoid any confounds with risk. Future work should focus on higher effort levels and potentially even go beyond 100% MVC to clarify this issue. While 100% MVC is clearly the maximum a participant can produce, efforts could be made harder without involving risk, for example by involving both hands or legs. Another point worth noting is that the model we propose here converges to zero. We speculate that with increasingly high effort levels, there may be a point where participants would be willing to pay money to avoid having to exert effort, or would reject choices with exceedingly high costs if given the option, which would imply a negative value. Addressing this question would require a different experimental design. For example, one could use a fixed value for the effortful option and a variable value for the non-effortful option, which would also allow distinguishing additive from multiplicative discounting. Though negative values are not currently incorporated in our model, we speculate that an improved effort discounting model that can accommodate negative effort values will still entail a turning point, rather than converging to –∞ in the way a quadratic function does. The remaining question then is whether, or under which conditions, this may occur. The implication of an asymptote to a constant value would be that all efforts which are unattainable (i.e. too large to be realized) affect value in a similar way. We hope that future work will address these remaining questions.

### Generalization of the proposed model for effort discounting

In order to avoid confounding influences between effort and delay, we manipulated effort such that higher levels required a stronger muscle force over a fixed period of time. This type of effort stands in contrast to persistent efforts, which require repeated execution of the same movement (walking up a staircase; pressing a lever), and which are commonly used in rodent experiments [68–71]. It is conceivable that our characterization of the discount curve is specific to situations where effort and muscle force are in direct relation with each other (e.g., deciding to hit the break at a yellow light, lifting a water container etc.). The persistent efforts required on fixed-ratio schedules share some features with the force-related effort used here in that the harder conditions may lead to exhaustion and temporary muscle fatigue. However, persistent efforts also share a critical feature with delay discounting in that more difficult efforts (e.g. more lever presses), require additional time to be completed. This causes an additional delay to reward delivery, which of course complicates the interpretation of how effort and delay independently influence choice. It is an intriguing hypothesis that the linear discounting of efforts suggested in the context of fixed-ratio schedules [37] may be caused by an interaction of simultaneously occurring discounting processes, namely the convex discounting of delays, and the concave discounting of force-related effort put forward here.

Our second finding shows that the steepness of effort discounting relates to the overall range of rewards at stake. Our hypothesis was that the same energetic requirement will affect larger rewards less than smaller rewards, because we might be willing to work harder for a better outcome. This ‘magnitude effect’ is known for delay costs, where different discount rates best explain choices in different reward ranges [59,61–64]. Importantly, it describes a relative rather than an absolute decrease in value. Our data provides support for this hypothesis also for effort costs, as reflected in a stronger decrease in value for smaller reward ranges, and a shallower discount curve for larger reward ranges. Critically, in our two experiments effort discounting was consistently concave, and the reward range affected the scale but not the shape of discounting. This implies that the model established here as the best model for effort discounting may generalize to different ranges of magnitude than the ones used here.

A large body of literature similarly shows that the range of delays tested (from seconds to days to years) has an impact on the discount rate. However, the hyperbolic nature of temporal discounting is remarkably consistent across all time scales [72–77]. Whether this generalization also holds true for a broad spectrum of effort ranges and the sigmoidal model for effort discounting proposed here remains to be tested. In any case, since the fitted parameters depend on the range of reward magnitudes used, separate fits need to be obtained for ranges of rewards in different orders of magnitude, which could be seen as a limitation of both models.

### No relationship between effort and delay discount tendencies

The behavioral dissociation between effort and delay discounting described here is consistent with the notion that these two decision costs may be supported by different neural networks. Although effort and delays are commonly referred to as decision costs, one attractive proposal is that while effort is a decision cost that is ascribed to a particular action, delay is one of the parameters (like risk, uncertainty) that can be ascribed to—and discount—the value of an outcome ([43], but see [44,45] for how delays can relate to actions). Consistent with this idea, we are not aware of any evidence to suggest that delay- and effort-discounting behaviors correlate, and indeed there is no evidence for a relationship between the individual discount tendencies for effort and delay costs in our data. This means, a person who is willing to wait for reward does not necessarily also accept high levels of energy expenditure to secure reward and vice versa; in line with this, these abilities have developed independently in different species [6]. Furthermore, lesions of ACC impair effort-guided choices, but leave intact those guided by delay, while lesions of OFC have the opposite effects, suggesting that (at least partially) distinct networks of brain regions support these two functions [46,78]. This evidence is consistent with the deficits seen in patients, where dysfunction of ACC-related circuits causes symptoms such as apathy [32], while damage to OFC-related circuits causes impulsivity and disinhibition [46,79–81]. The ACC may be particularly crucial for guiding effort-based choices because neurons encode both the effort cost and reward payoff of an option as well as the selected action [25,82–84], and also the number of actions required to obtain reward [85]. There is also some evidence that different neurotransmitters are critical in assessing delay- versus effort-related costs [86,87], and that dopamine manipulations have differential effects on these two behaviors [69].

### Potential applications

Our data and modeling approach provide an important step forward in understanding how effort influences the decision making process. The behavioral model proposed for effort discounting here may provide a powerful diagnostic tool for quantifying motivational disturbances evident in clinical disorders (i.e., apathy, depression, fatigue, abulia), and may thus be critical in understanding the basis of several common psychiatric and neurological conditions [9,88]. The motivational disturbances experienced in some of these conditions can profoundly affect one’s personal and professional life, and cause limitations in terms of life function, interaction with the environment, and responsiveness to treatment [26,89,90]. Understanding how reinforcement influences the willingness to exert effort may also be of relevance for neurorehabilitation following brain injury which is commonly based on effortful and repeated physical exercise. In summary, as well as contributing to the understanding of clinical disorders involving motivational disturbances, the formal mathematical framework provided here will provide a powerful tool for the neural and behavioral study of effort-based choice.

## Materials and Methods

### Ethics statement

This study was ethically approved by the UCL Research Ethics committee, and all participants gave written informed consent.

### Participants

Twenty-eight (Experiment 1: age range: 22–35 years, mean age 26 years, 20 female, 8 male) and fifteen (Experiment 2: age range: 22–35 years, mean age 26 years, 11 female, 4 male, including the same fifteen as in Experiment 1) right-handed volunteers with no history of neurological or psychiatric disorder, and with normal or corrected-to-normal vision, participated in this study. Five participants from Experiment 1 and one participant from Experiment 2 were excluded from the analysis (see below). Inclusion in Experiment 2 was a self-selection process; every participant from Experiment 1 was invited to take part and all those available completed Experiment 2.

### Experimental task and procedure

#### Experiment 1: Dissociating delay and effort discounting

All participants completed two versions of the task on separate days and in a counterbalanced order, with choices being made between two delayed or two effort-demanding monetary outcomes respectively (average days between sessions: 10±3; Fig. 1A). Participants were seated comfortably in front of a computer monitor and held a custom-made grip device in each hand. To account for individual differences in grip strength, a grip calibration was performed at the beginning of the session. Each participant’s baseline (no grip) and maximum right-hand voluntary contraction (MVC) were measured once over a time window of 3s, independently for both hands. The maximum on the thermometer was set to 80% MVC (this is referred to as 100% in the actual experiment and analyses).

Participants received both written and oral task instructions, and performed ten force grips (4s) with both hands followed by 15 practice trials for familiarization with the effort levels (for the effort session), and the task respectively. During familiarization, the force grips covered the entire range of grip forces used in the main experiment, in a randomized order. This enabled participants to experience force levels before being asked to make choices involving these force grips.

The main experiment consisted of two blocks of 100 choices each. Participants made choices between two options presented to the left and right of fixation; both options were associated with varying reward magnitudes and costs (S5A Fig.). The reward magnitude was shown as a number between 0 and 75, corresponding to pence and pounds in the effort and delay versions of the task, respectively; the cost was shown as a horizontal bar, indicating the required grip strength, or the number of weeks until reward payout (between 0 and 75), respectively (Fig. 1).

Because participants held a grip device in both hands, choices were indicated by a brief squeeze of the gripper of the corresponding hand rather than using a button press (max. response time: 5s; required level: 35% of MVC), upon which the unchosen option disappeared. Note that until this point, trials were identical for the two task versions. In the delay task, this was the end of the trial (ITI: 1–3s).

To avoid fatigue, on the effort task, effort choices were only realized on an unpredictable 30% of trials (∼15% per hand; Fig. 1). A thermometer appeared on the screen with the required force level corresponding to that of the chosen option, and indicating that a power grip of 12s was required with the corresponding hand. Participants were given on-line feedback about the applied force level using changing fluid levels in the thermometer. On successful application of (at least) the required force for 80% of the time, a green tick appeared (700ms) and the reward magnitude of the chosen option was added to the total winnings. Otherwise, the total winnings remained unchanged (red cross: 700ms). On the remaining two-thirds of trials, the message ‘no force’ appeared for 700ms, and the next trial commenced (ITI: 2–4s).

#### Experiment 2

The basic experimental procedure of both sessions was identical to Experiment 1 except that this time only the right hand held a grip-device because the left option was associated with a fixed effort level of zero. The left hand rested on the keyboard. The force calibration was therefore only performed for the right hand, but now three times and for a duration of 12s. Average values of all three calibrations were used to define the participant’s individual force range (0–100%) used in the main experiment. Analysis of calibration results from this experiment showed that the average of three calibrations did not differ from the MVC obtained in the first calibration (p = 0.16), showing that the calibration procedure used in Experiment 1 was valid.

Before the start of the experiment, participants received written and oral instructions, completed ten practice force grips and 15 practice trials, as in Experiment 1. Visual display and task timings were identical in both Experiments unless indicated otherwise.

The main part of the experiment involved two blocks of 96 trials of a choice task (**Fig. 1B**). Choices were made between a free reward of 40p (block1) or £2 (block2) presented on the left, and an effort-demanding reward with changing reward magnitude and effort level, presented on the right. As before, the reward magnitude was shown as a number and required force levels were indicated as the height of a horizontal bar. Choices were indicated with the left hand using two buttons on a keyboard (max RT: 5s).

The alternative option could take one of six possible effort levels ([15 25 35 50 75 100]% MVC). The reward magnitude associated with each force level was adjusted on a trial-by-trial basis using an adaptive staircase algorithm, independently for each effort level (Parameter Estimation by Sequential Testing, PEST, see [91]). The staircase procedure allowed for determining the point of subjective indifference at which participants assign equal value to both options. Because the trial order was randomized for the six effort levels, six separate staircases were interleaved. None of the subjects was able to detect these rules used for adjusting rewards, as assessed verbally upon completion of the experiment. The staircase was initialized with a reward magnitude of 60p±6 (block1) or £2.6±0.2 (block2), and it was increased or decreased using an initial step size of 8p (block1) or 24p (block2), depending on whether participants rejected or accepted to take the alternative option. The staircase was re-initialized when the step size had reached a fourth of its original size (block1: 2p; block2: 6p). For details refer to the PEST procedure in [91]. Because in most cases, the reward magnitude of the effortful option was higher than that of the default option, we also refer to them as the higher-reward/higher-cost (HRHC) and lower-reward/lower-cost (LRLC) options.

On 85% of trials, no effort was required (“no force”, 700ms; winnings unchanged; ITI: 2–4s). By contrast, on an unpredictable 15% of trials, if the effortful option had been chosen the force grip had to be realized with the right hand. Online feedback via the thermometer, feedback upon completion of the 12s, and updates of total winnings were provided as in Experiment 1. If, however, participants had chosen the default option, they had to wait for 12s without producing any force, and the default magnitude was added to the total winnings. This ensured that effort costs were unconfounded from temporal delays.

In both Experiments 1 and 2, to ensure choices were independent and not driven by fatigue, participants only performed the effort on a subset of trials but importantly, they did not know at the time of choice whether this trial would be real or hypothetical. Thus, each choice had to be treated as potentially real, and thus relevant to the final payment. Furthermore, following each 12s-effort, the ITI was prolonged by 3.5 seconds so that participants had sufficient time to recover before the next trial.

### Experimental stimuli

The choice stimuli were optimized for two different purposes in the two Experiments. In Experiment 1, choice options were identical for every individual and for the effort and delay versions of the task. They were chosen such that they would maximally contribute to differentiating between three competing behavioral models, e.g. so that an agent with hyperbolic discounting would make different choices to someone exhibiting linear or concave discounting behavior (for each of the three models, we modelled nine agents, spanning from shallow to steep, and selected stimuli that contributed in at least two simulated agents to distinguish at least two of the models). Second, the stimuli in Experiment 1 covered the entire range of magnitudes and costs, and magnitude and cost differences (S5A Fig.), and were paired such that different levels of (the smaller) magnitude were paired with a wide range of cost differences. We did not include trivial choices where a small cost difference would be paired with a large magnitude difference because we expected participants would be entirely magnitude driven on such a trial and it would therefore be uninformative with regards to their discount behavior. We also kept the number of trials with two high-costs options small because we did not want to force ‘effort-averse’ participants into having to exert a high force. In Experiment 2, by contrast, choice options were adjusted based on individual participant’s preferences in order to obtain points of subjective indifference. Therefore, the number of effort levels had to be restricted in this Experiment.

### Recordings of grip strength

The grippers were custom-made and consisted of two force transducers (FSG15N1A, Honeywell, NJ, USA) placed between two moulded plastic bars [92]. A continuous recording of the differential voltage signal, proportional to the exerted force, was acquired, fed into a signal conditioner (CED 1902, Cambridge Electronic Design, Cambridge, UK), digitized (CED 1401, Cambridge Electronic Design, Cambridge, UK) and fed into the computer running the stimulus presentation using Cogent (http://www.vislab.ucl.ac.uk/cogent_graphics.php). This enabled us, during effort trials, to give online feedback reflecting the exerted force using the thermometer display.

### Choice of force range

The range of forces and the duration of the grip on effort trials (12s) had been determined in pilot experiments. Our aim was to use force levels that are factored into the choice process, but that are still in the range of possible forces for a given participant. This seemed most like the type of choices we make in real-world scenarios. Pilot experiments had shown that durations significantly shorter than 12s meant that participants often ignored the effort because they were merely interested in earning money. Importantly, the duration of 12s was fixed and unrelated to the force level, thus enabling us to study effort unconfounded from temporal costs.

### Analysis of grip traces

The force traces obtained from realized trials were analyzed to establish the time it took participants to reach the required force level, the time (out of 12s) that they spent at the required level, and the mean and variance of the produced force output (Fig. 2). First, every time point was classified as above the required level or below the required level [alternatively: within +0.2 of the required level] and the time to reach the criterion was defined as the first time at which the effort level was reached, provided the 10 subsequent time points (170ms) also exceeded the required level. For Experiment 1, it was more appropriate to report the time at which they had reached the required level, but were at most 20% above the required level (also for 170ms) because a grip force of 0.35 was exerted at the time of response when the 12s grip started, implying that the required force level was already exceeded at time zero for lower forces, which would result in a misleading RT of 0ms (see Fig. 2C). Force traces were then grouped into six bins (equally-sized between [0,1] in Experiment 1, and pre-defined as [15 25 35 50 75 100] in Experiment 2) to display average force time courses (Fig. 2C,D), and the variance of the force output across the 12s interval was measured and averaged across all trials in the same bin (Fig. 2E,F). Finally, we tested for a correlation between the required and produced force levels using the mean force produced between 2–10s from every effort trial (Fig. 2G,H).

### Payment

In the effort experiments participants were paid the sum of the rewards accumulated on all effort trials (Experiment 1: average: £21.40; range: £15.70-£25.75; Experiment 2: average: £35.62; block1: £6.04; block2: £29.59). In the delay experiment, they were paid out one choice from a pseudo-randomly selected trial and the money was transferred via bank transfer, pre-dated to the corresponding delay (average: £21.60 in 8 weeks). This procedure was chosen for several reasons. First, while paying out one randomly selected option is an established procedure for delay-based choices, we believe efforts would not have had a strong impact on choice behavior if we had kept them abstract and only realized one at the end of the experiment. Participants knew that effort levels were within the range they could achieve, and the majority would have chosen the HRHC option in all trials to ensure a larger reward outcome, knowing only one effort needed to be produced at the end. Second, the evaluation of effort levels depends heavily on the bodily state of a person, and the occasional experience of an effort meant that its evaluation was more real.

Given the different number of realized trials in the effort and delay sessions of Experiment 1, we chose different ranges of reward (pence and pounds, respectively) and thus approximately matched the overall reward sum. Extensive piloting had shown that larger reward ranges would mostly produce choices of the HRHC option for effort (and vice versa, smaller rewards for delay would have led to choices of the immediate option on most trials). Of course, to match the reward range, we could have instead increased the difficulty of the required efforts, but given practical and time constraints, it seemed reasonable to adjust the reward range so that both types of costs would influence choices to a similar extent which we achieved (54% and 52% HRHC choices in effort versus delay).

### Exclusion of participants

#### Experiment 1

Because the main aim of this study was to investigate effort discounting behavior, it was critical that participants considered the required effort level for their choices. Therefore, three participants who chose the lower-reward/lower-effort option on five trials or less (i.e., > = 97.5% high-effort choices) were excluded from the analyses. Two additional participants were excluded from the analyses: one did not understand the task instructions (hoping throughout the experiment that trials would be hypothetical), and one subsequently reported nerve damage in the left hand.

#### Experiment 2

One participant was excluded because of a highly inconsistent (‘random’) choice pattern. Because of this, the staircase only converged for 2 out of 12 (2 blocks x 6 levels) effort levels, and produced negative offered reward magnitudes.

### Behavioral models (Experiment 1)

Our main analyses regarded participant’s choice behavior, but a detailed analysis of response times and a logistic regression of choices were also performed (see S1 Text; S1 Fig.). We compared several models to formally characterize effort discounting behavior.

#### Hyperbolic

Temporal delay devalues rewards hyperbolically across time [10–12], but it has been suggested that this might also be true for effort [29,39]. The hyperbolic model predicts that additional effort costs have a large impact on discounting value when added to small effort costs but a small impact when added to high effort costs (Fig. 3A):

$$V=\frac{M}{\left(1+kC\right)}$$

Here, *V* denotes the subjective value, *M* denotes the reward magnitude, *C* the cost (effort or delay) and *k* the discount factor which is the only free parameter that is fitted. We hypothesized that this model would perform best in describing discounting behavior for the choices involving delay, but would not provide a good explanation of effort-based choices.

#### Linear

The second model previously suggested to describe effort discounting [37] is a simple linear model, which implies a constant integration of effort independent of reward amount, i.e., an additional fixed cost Δ*C* devalues a reward by the same amount, regardless of whether it is added to a small or a large pre-existing effort level:

V=M−kC

The parameter *k* describes the slope and is estimated. This model has been suggested in the context of effort-based choice behavior when persistent effort has to be made over time (i.e., repeated lever presses)[37].

#### Quadratic

Next, we included a quadratic model [40] in which rewards are devalued by an increasing amount with growing effort/cost levels, according to a power function:

$$V=M-kC^{2}$$

Here, the parameter *k* describes the curvature and thus, the steepness of the power decrease. This model converges to -∞ with increasingly large efforts.

#### Sigmoidal

Our two main criteria to define a theoretically grounded model for effort discounting were (1) that it can take initially concave shapes because (a) intuition tells us that small efforts should have a relatively small or no impact on value, but large efforts should lead to steep discounting of rewards; (b) studies on the perceived ‘sense of effort’ at different force levels show a reduced sensitivity to lower compared to higher efforts, and that the perceived sense of effort increases as a power function of the exerted force level [41,42]; and (2) that it becomes increasingly less steep for efforts that are unattainable, thus entailing a turning point. We developed a model to fulfill our two main criteria, and describe its components step by step. First, by subtracting a sigmoidal function from *M*, we can get a function with roughly the desired shape:

$$V=M\left(1-\frac{1}{1+e^{-k\left(C-p\right)}}\right)$$

However, this model does not go through (*C*,*V*) = (0,*M*), i.e., even without costs (cost of 0), there will already be decreased devaluation of the reward. Therefore, a constant corresponding to the subtracted term at *C* = 0 (1/(1+exp(kp)), was added to force the model through (*C*,*V*) = (0,*M*):

$$V=M\left(1-\frac{1}{1+e^{-k\left(C-p\right)}}+\frac{1}{1+e^{kp}}\right)$$

This adjustment has one unwanted effect because the model will now converge to 1/(1+exp(kp)). Thinking of discounting as a relative decrease in value (multiplicative), and in the context of overall positive values, the smallest value that could be reached would be 0% of the original value. Therefore, the final adjustment here was to multiply the subtracted term with (1 + 1/exp(kp)), resulting in a function that converges to zero. However, future work needs to test this assumption directly. It could be expected that subjective discounted value can go negative, which would require an adjustment of our proposed model, and it is also unclear whether effort discounting is additive or multiplicative in nature. Here our final model (Fig. 3A) is given by:

$$V=M\left(1-\left(\frac{1}{1+e^{-k\left(C-p\right)}}-\frac{1}{1+e^{kp}}\right)\left(1+\frac{1}{e^{kp}}\right)\right)$$

The two fitted parameters are the slope *k* and the turning point *p*.

#### Flexible power function

For the purpose of a control analysis, we also included a two-parameter power function, which matched the number of parameters of the sigmoidal model, but whose features were more similar to the quadratic model proposed previously [40]. This model was included to verify whether the sigmoidal model may provide a better description of the data due to its higher flexibility in the lower range of efforts which was also present in this model. It was described as

$$V=M-kC^{p}$$

For all models, the softmax function was used to transform the subjective values *V*_*1*_ and *V*_*2*_ of the two options offered on each trial into the probability of choosing option 1.

$$P\left(\mathrm{chooseOption}1\right)=\frac{1}{1+e^{-\beta \left(V1-V2\right)}}$$

The temperature *β* determines the steepness of the softmax function, and thus how sensitive the choice probability is to differences in subjective value between *V*_*1*_ and *V*_*2*_.

### Bayesian parameter estimation and model comparison

For all models, the free parameters (*k*, *β*) or (*k*, *p*, *β*), respectively, were fitted using the Variational Laplace algorithm [93,94]. This is a Bayesian estimation method which incorporates Gaussian priors over model parameters and uses a Gaussian approximation to the posterior density. The parameters of the posterior are iteratively updated using an adaptive step size, gradient ascent approach. Importantly, the algorithm also provides the free energy *F*, which is an approximation to the model evidence. The model evidence is the probability of obtaining the observed choice data, given the model. This free energy approach yields better model scores than does the Akaike Information Criterion (AIC) or Bayesian Information Criterion (BIC) [95].

The model evidence can be decomposed into an accuracy and a complexity term. The accuracy term reflects the model fit to the current data sample, whereas the complexity term penalizes models that have unlikely parameter values. The combination of the two terms provides a Bayes optimal estimate of how good a model is and is the standard criterion used for model comparison in the Bayesian statistics literature [96,97]. Other metrics for model assessment include the use of out-of-sample model fit using for example cross–validation. There is a high degree of correlation between findings from cross-validation and Bayesian model comparison (see for example [98]).

In Experiment 1, the log model evidences averaged over subjects for the effort data were −95.3 for the sigmoidal model and-130.6 for the hyperbolic model, providing a difference of 35.3 in favor of the sigmoidal model. As differences of log model evidence greater than 5 provide ‘very strong’ evidence for a hypothesis [99,100] it is clear that the sigmoidal model is superior. Breaking down the log model evidences into contributions from accuracy and complexity [99] we found that the sigmoidal model had an accuracy of −85.6 and a complexity of 9.7 whereas the hyperbolic model had an accuracy of −124.8 and a complexity of 5.8 (see S2 Table). Note that log model evidence = accuracy—complexity (e.g., -95.3 = -85.6–9.7). Thus, the sigmoidal model was more complex but this additional complexity was more than compensated for by an increase in accuracy.

For the delay data, the log model evidences averaged over subjects were −159.2 for the sigmoidal model and −156.4 for the hyperbolic model, providing a difference of 2.8 in favor of the hyperbolic model. Breaking down the log model evidences into contributions from accuracy and complexity we found that the sigmoidal model had an accuracy of −153.6 and a complexity of 5.7 whereas the hyperbolic model had an accuracy of −151.4 and a complexity of 5.0 (see S2 Table). Thus, the models were of similar complexity but the hyperbolic model more accurately described the data. Readers more familiar with model selection criteria such as AIC and BIC may be surprised here as these latter criteria have model complexity terms that scale in proportion to the number of parameters. Thus models with more parameters are always more ‘complex’. However, the Bayesian model evidence penalizes models in proportion to how far the posterior is from the prior (as quantified by the KL-divergence). Thus, in the limit of the posterior equaling the prior, our beliefs about model parameters will not change and the penalty will be zero. This property renders the Bayesian model evidence a better model comparison criterion than AIC or BIC [95].

To maximize our chances to find global, rather than local maxima using the gradient ascent algorithm, parameter estimation was repeated over a grid of initialization values. The grids contained eight initializations per parameter, spanning the relevant parameter range. The optimal set of parameters, i.e., that obtained from the initialization that resulted in the maximal free energy, is reported in this manuscript.

The above computation of the model evidence is dependent on the choice of prior. To ensure robustness of our findings, the above estimation was therefore repeated with the prior covariances set to be an order of magnitude larger and smaller, respectively. Changing the prior variances in this way had no effect on any of our conclusions. We can therefore be confident our conclusions are driven by the behavioral data rather than the prior beliefs.

The free energy *F* obtained for each model and participant was then used to perform a formal Bayesian Model Comparison (BMC) at the group level [101,102]. These group level inferences provide an estimate of the frequency with which a model occurs in the population from which the participants are drawn. For the *i*th model, r_*i*_ is the frequency with which it appears in the population. The BMC approach uses the table of model evidence values (subjects x models; see S2 Table) to estimate a posterior distribution over r_*i*_. The mean of this posterior distribution, mp(*i*), is our best estimate of *r*_*i*_. We can additionally ask about the probability that model *i* occurs the most frequently. This is known as the exceedance probability, xp(*i*).

BMC at the group level, also known as random effects model [101,102], has become a standard statistical test in the fields of neuroimaging and behavioral modeling ([101] cited > 250 times). As described above, the central quantity of interest is r_*i*_, the frequency with which model *i* is used in the population from which the subjects are drawn. Given a table of model evidence values (see S2 Table) an algorithm [101,102] can be derived for computing a posterior distribution over r_i_, from which subsequent inferences can be made. Intuitively, this is based on two quantities (1) the proportion of subjects in the sample group that favor model *i* and (2) the degree to which the models are favored. As we will see in the results section ${\hat{r}}_{1}=0.80$ for the delay task (80% subjects in the population use the hyperbolic model) and ${\hat{r}}_{2}=0.96$ for the effort task (96% subjects use the sigmoidal model; see Materials and Methods and Results).

Our main analysis asked whether the hyperbolic, linear, quadratic or sigmoidal models were better at describing participants’ choices. An important feature of BMC is that it can compare models with different numbers of parameters in an unbiased manner (see above).

Experiment 1 was optimized to establish a direct dissociation between delay and effort-based choices, but was less sensitive to slight changes in model shapes. Our main analysis was therefore a comparison between the sigmoidal model developed above (initially concave shape but convex after a turning point) and the hyperbolic model proposed for delay discounting [10–12]. In a second step, to confirm that the concave feature of the model constitutes a critical improvement for modelling effort discounting, we performed pairwise comparisons between the hyperbolic model and the two other concave alternatives (quadratic and two-parameter power function). Finally, we included all models in the same model comparison to check which discount function best described effort discounting in our task.

### Using utility instead of reward magnitude

Rather than using reward magnitudes on a scale of pounds and pence, in economic choice contexts the utility is often used as a more subjective measure of the experience of a monetary reward. To test whether the shape of discounting would change when utility was used instead of reward magnitude, the magnitude M was replaced by M^α^ in all models described above. Because our stimuli were not optimized for fitting this additional parameter, we used a generic utility value of α = 0.8. This value provides an estimate for the average utility parameter of healthy human participants [57,58] and if anything slightly overestimates the curvature of the utility function. Therefore, it provides a conservative test of the effect of using utility on the resulting discounting function. Parameter estimation and model comparisons were repeated for all models using this generic measure of utility.

### Fitting indifference points (Experiment 2)

The advantage of keeping one option constant in Experiment 2 was that stimuli only varied along two dimensions (one effort and one reward magnitude). As a result, choice preferences could be easily visualized (Fig. 4A,C) and indifference points determined, free of any assumptions about the precise discounting function. By contrast, Experiment 1 allowed us to test a wide range of effort and magnitude differences (S5A Fig.), and allowed for a more robust characterization of choice preferences (because stimuli varied along four dimensions). Because understanding choice preferences in Experiment 1 relied on the use of behavioral models, we now conducted a virtually model-free analysis of effort discounting. To this end, we determined points of subjective indifference based on participants’ choices. The sixteen choices performed for each of the six levels of effort were plotted as a function of the magnitude of the alternative offer (Fig. 4B). A simple sigmoid was then fitted using equation (1) below, and the indifference point was defined as the reward magnitude (on x) at which the sigmoid crossed y = 0.5, which corresponds to α; β is the slope. This procedure is illustrated in Fig. 4B.

$$\frac{1}{1+e^{-\beta \left(x-\alpha \right)}}$$

## Supporting Information

S1 Text The supplementary methods and results report an analysis of response time and choice based on simple regression analyses, and include additional tables reporting model parameter estimates, accuracy and complexity terms, and results of control analyses. (DOCX)

S1 Fig Response times and choices are influenced by costs and benefits (Experiment 1). **A,** Response times are shown as a function of the difference in reward magnitude between the two choice options, and the sum of the costs of the two choice options, separately for effort costs (left) and delay costs (right). While large reward differences speed up the choice process, larger overall costs slow response times. This effect is observed independent of the type of cost. **B,** Mean (± SEM) parameter estimates from a logistic regression analysis of each participant’s choice pattern. Participants’ choices were driven by both options’ reward magnitude and cost level, showing that all dimensions of the outcomes were taken into consideration for computing a choice. Benefits and costs had opposite effects: larger costs discouraged and larger reward magnitudes encouraged the choice of an option.
(TIF)

S2 Fig Winning models for effort and delay tasks with all models (Experiment 1). **A-B**, Bayesian Model Comparison comparing all four (**A**) or five (**B**) behavioral models: in both comparisons, the hyperbolic model provides the best explanation for choices on the delay task. In the effort task, the other concave models (quadratic in **A** and quadratic + two-parameter power function in **B**) provide an almost comparable explanation of choices as the sigmoidal model. This is because choice stimuli were not individually adjusted and optimized to distinguish similar discounting shapes, but instead designed to distinguish hyperbolic and concave discounting behaviors. **C**, The individual and average fits obtained from a two-parameter flexible power function show that this model entails a steep decline towards negative values. This model has less evidence than the sigmoidal model despite having the same number of parameters and similar flexibility in the lower range of efforts. **D**, Sigmoidal fit to delay choices shows that the same model produces an opposite shape in the delay compared to the effort version of the task (compare Fig. 3C).
(TIF)

S3 Fig Percentage of predicted choices (Experiment 1). **A**, Percentage of correctly predicted choices for the hyperbolic and sigmoidal models. Note that the percentage of correctly predicted choices does not take into account the additional model parameter of the sigmoidal model, which importantly was considered in the formal model comparison results shown in Fig. 3B. **B**, Percentage of correctly predicted choices in a subset of 14 of 23 participants who in addition to the original delay task (with bars signaling the delay, as for effort) also completed a version that indicated delays using words. In the latter task, we were able to predict a much higher percentage of choices. All error bars/vertical bars denote SEM.
(TIF)

S4 Fig Sigmoidal fits using produced instead of required force. The individual and group fits of the winning sigmoidal model for Experiment 1 are shown as in Fig. 3C (left), but here, instead of using the required force, analyses were based on the force level actually produced in a given trial (i.e., %MVC here refers to the *produced* effort level). However, because not all trials involved an effort production, the ‘produced’ effort had to be predicted for all trials (*predicted produced*). This was achieved by fitting the force from all effort production trials (see Fig. 2G-H) using a quadratic trend, to predict the force given the trial’s required force level. Overall, the ‘predicted produced’ force slightly exceeded the required force and increased somewhat supra-linearly for higher efforts (see Fig. 2, G-H). However, and importantly, the resulting sigmoidal fits from this analysis were qualitatively similar to the original analysis, suggesting that using the predicted produced force does not change any of the conclusions.
(TIF)

S5 Fig Choice stimuli (Experiment 1). **A**, The choice stimuli were carefully designed to cover the entire range of magnitudes and costs, and magnitude and cost differences. They were also optimized for distinguishing between concave and convex behavioral models. **B,** To illustrate that the percentage of choices explained by a model depends on the offered choice stimuli (see Results), we repeated the model fitting and logistic regression analysis on the highlighted subset of trials which was defined as choices in which (a) both options were in the lower cost range, with at least one option with cost <0.2, and the difference to the second option not higher than 0.3, or (b) both options were in the higher range of cost levels (both costs>0.4). This included 25 choices for the lower and 14 choices for the higher cost range. **C**, The percentage of choices of the higher-reward/higher-cost (HRHC) option was higher in the subset of trials with small efforts compared to large efforts. **D**, Furthermore, reward magnitude (blue bars) influenced choices less on these high cost (right), compared to low cost (center) trials.
(TIF)

S1 Table Individual parameter estimates (Experiment 1). For each subject in Experiment 1, the table shows the parameter estimates for the hyperbolic (k) and sigmoidal model (k and *p*; see fits in **Fig. 3C**).
(DOCX)

S2 Table Accuracy, complexity, and log-evidence (Experiment 1). Accuracy, complexity and log-evidence values estimated for each participant and model. These values determine the results of the Bayesian model comparison shown in Fig. 3B (Experiment 1). Note that log model evidence = accuracy—complexity
(DOCX)

S3 Table Exerted versus required force (Experiment 1). Our main analysis reported in the manuscript was based on the required force level. The table shows the result of a control analysis which used the produced (‘predicted produced’) rather than required force level for Bayesian parameter estimation and model comparison (mp = mean posterior; xp = exceedance probability, values refer to models 1–2 in Experiment 1). The produced force was predicted from a quadratic model fitted to each participant’s force produced in effort production trials. None of the conclusions change with this analysis, showing that the concave shape of effort discounting is not due to slight increases in the produced force and its variability observed with increasing effort levels.
(DOCX)

S4 Table Percentage of high-cost choices (Experiment 1). Experiment 1. **Left:** The percentage of higher-reward/higher-cost choices on trials following force-production trials, compared to an equally large subset of trials following a no-effort trial. **Right:** Percentage of HRHC choices on trials following an easy compared to a hard realized effort (median split). The absence of statistical differences shows that choice behavior was not affected by local trial-by-trial fatigue.
(DOCX)
