# Correction: Behavioral Modeling of Human Choices Reveals Dissociable Effects of Physical Effort and Temporal Delay on Reward Devaluation

**DOI:** 10.1371/journal.pcbi.1005401

**Published:** 2017-02-27

**Authors:** The PLOS Computational Biology Staff

## Notice of Republication

This article was republished on April 14, 2015 to correct errors in S1 Text and S1-4 Tables. Please download these files again to view them correctly.
