# Supplementary material for: Behavioral Modeling of Human Choices Reveals Dissociable Effects of Physical Effort and Temporal Delay on Reward Devaluation
Source: PLoS Comput Biol. 2015 Mar 27;11(3):e1004116. doi: 10.1371/journal.pcbi.1004116 (PMC4376637; doi:10.1371/journal.pcbi.1004116)
Supplement: S1 Text — (DOCX) [file pcbi.1004116.s001.docx]

**Supporting Information – S1 Text**

**S1 Materials and Methods**

***Explaining response times with value, reward, and cost***

To test whether response times (RT) in Experiment 1 were driven by the subjective values of the offers, offered reward magnitudes, or costs (i.e., effort or delay), we fitted several linear regression models to the RT data. All models contained a constant term and a regressor for response hand, and all models using data from the effort task also included a regressor indicating whether or not effort was exerted on the previous trial. The first model examined whether RTs relate to decision difficulty, or the overall value of the offers. Therefore, the difference and the sum of the subjective values of the offers were included as two additional regressors (median absolute correlation: 0.10 ± 0.04). The second model contained the difference between the offered magnitudes and between the offered costs (correlation: 0.25) as the two regressors of interest, and the third model the sum of magnitudes and the sum of costs (correlation: 0.16). Finally, to establish whether costs and rewards were interacting with RTs independently of decision difficulty, we tested four additional models, where value difference was combined with one of magnitude difference, magnitude sum, cost difference and cost sum, respectively (correlations: 0.63, 0.13, 0.13, 0.12).

***Logistic regression of choice data***

To examine which task variables affected choice behavior, a logistic regression was fitted to participants’ choices (1=RH, 0=LH) using the following seven regressors for the effort task: a RH-LH bias (constant term); 12s-effort with the LH on previous trial; 12s-effort with the RH on previous trial; reward magnitude left; reward magnitude right; effort left; effort right; and the same five regressors but without the ‘12s-effort with LH/RH on previous trial’ for the delay task. T-tests performed across participants on the obtained regression coefficients were adjusted for multiple comparisons, using Bonferroni correction.

This analysis was repeated on a subset of trials to test whether magnitude was taken into consideration in the same way on high and low cost trials (see **S5 Fig**, B). We also compared how often participants chose the higher-reward/higher-cost (HRHC) option in these two subsets of trials. The subsets of trials were chosen to be at the low and high end of the cost-spectrum, i.e., when both options involved low cost levels, or when both options involved high cost levels: (a) both options were in the lower cost range, with at least one option with cost <0.2, and the difference to the second option not higher than 0.3, or (b) both options were in the higher range of cost levels (both costs>0.4, **S5 Fig**). From our stimulus set, this included 25 choices for the lower and 14 choices for the higher cost range. This exploratory analysis was conducted *post-hoc*, without choice stimuli being optimized for this comparison. For example, we wanted to avoid forcing participants into high effort choice in case they were generally effort-averse, and therefore only a small subset of trials had both options in the high cost range. For this analysis, we chose a definition of cost ranges that meant the magnitude difference between choices in the two subsets was comparable, and the number of choices in each subset sufficient.

**S1 Results**

***RT results***

We tested whether RTs provided additional insight into the factors driving participant’s choice behavior. More specifically, we assessed whether choice RTs could be explained by the sum or difference of the model-estimated subjective values of the offers. Values were obtained from the respective winning models for both tasks. For both effort- and delay-based choices, RTs were strongly driven by the subjective value difference between the two offers, with larger value differences leading to significantly shorter RTs (effort: t(22) = -7.35, p = 2.35e-07; delay: t(22) = -6.36, p = 2.14e-06). The sum of the subjective values did not have such an effect (effort: p=0.67 and delay: p=0.66).

To examine the separate influence of costs and benefits on this RT effect, we repeated the analysis with effort, delay, or reward magnitude instead of subjective value entering the linear regression. The difference between the offered reward magnitudes had a significant effect on RTs (effort: t(22) = -3.19, p = 0.0042; delay: t(22) = -2.35, p = 0.028; **S1 Fig**), but the difference between efforts (p=0.31) and delays (p=0.93) did not. Interestingly, however, delay and effort impacted RTs in their sum, with an increasingly large cost total causing slower RTs (effort: t(22)=3.06, p= 5.7e-03; delay: t(22)=4.47, p= 1.91e-04). When overall higher forces or longer delays were involved, participants thus spent significantly more time deliberating over the options (**S1 Fig**). The sum of magnitudes had no consistent effect (effort: t(22)=-2.69, p=0.01; delay: p=0.21).

Finally, to establish whether the influence of magnitudes and costs was independent of decision difficulty, value-difference was included in the regression at the same time. Only the sum of the costs had an independent effect on RTs (effort: t(22)= 2.81, p=0.01; delay: t(22)=2.90, p=8.3e-03), but none of cost difference, magnitude difference or sum of magnitudes did (effort/delay: all p>0.15). Thus, overall levels of effort and delay had an effect on RTs that was independent of choice difficulty.

***Choice results***

Participants' choices were guided by the reward magnitude and cost of both options in both tasks (effort task: t_ML,22_ = -10.86; t_MR,22_ = 11.29; t_EL,22_ = 6.25; t_ER,22_ = -4.46; delay task: t_ML,22_ = -7.19; t_MR,22_ = 6.74; t_DL,22_ = 4.33; t_DR,22_ = -4.59; all p<0.001; **S1 Fig**, B). Importantly, when the right option was associated with a large reward magnitude, this encouraged a choice of the right option, while a high cost had the opposite effect and discouraged the choice of this option. There was no right/left hand choice bias, and for the effort task, choices were not influenced by whether an effort had been exerted with the right or left hand on the previous trial (all p>0.05). Thus, any influence of fatigue was negligible and choices between trials could be regarded as independent.

We performed the same logistic regression analysis separately on a subset of choices with two low or two high cost options to investigate how magnitudes influenced choice behavior in these trials. This showed that reward magnitude significantly influenced choices in the low cost trials (ML vs MR: t_22_ = -11.14, p<0.001) but only showed a trend-wise effect on choice in high cost trials (ML vs MR: t_22_ = -1.88, p=0.073; **S5 Fig**, D). Consistent with this, the percentage of high magnitude choices was larger in low compared to high cost trials (high cost: 81 ± 2%; low cost: 35 ± 6%; t_22_ = 8.18, p<0.001; **S5** **Fig,** C). Thus, reward magnitude had a smaller effect on choice when effort costs were high.
