# Supplementary figures and images for: Behavioral Modeling of Human Choices Reveals Dissociable Effects of Physical Effort and Temporal Delay on Reward Devaluation
Source: PLoS Comput Biol. 2015 Mar 27;11(3):e1004116. doi: 10.1371/journal.pcbi.1004116 (PMC4376637; doi:10.1371/journal.pcbi.1004116)

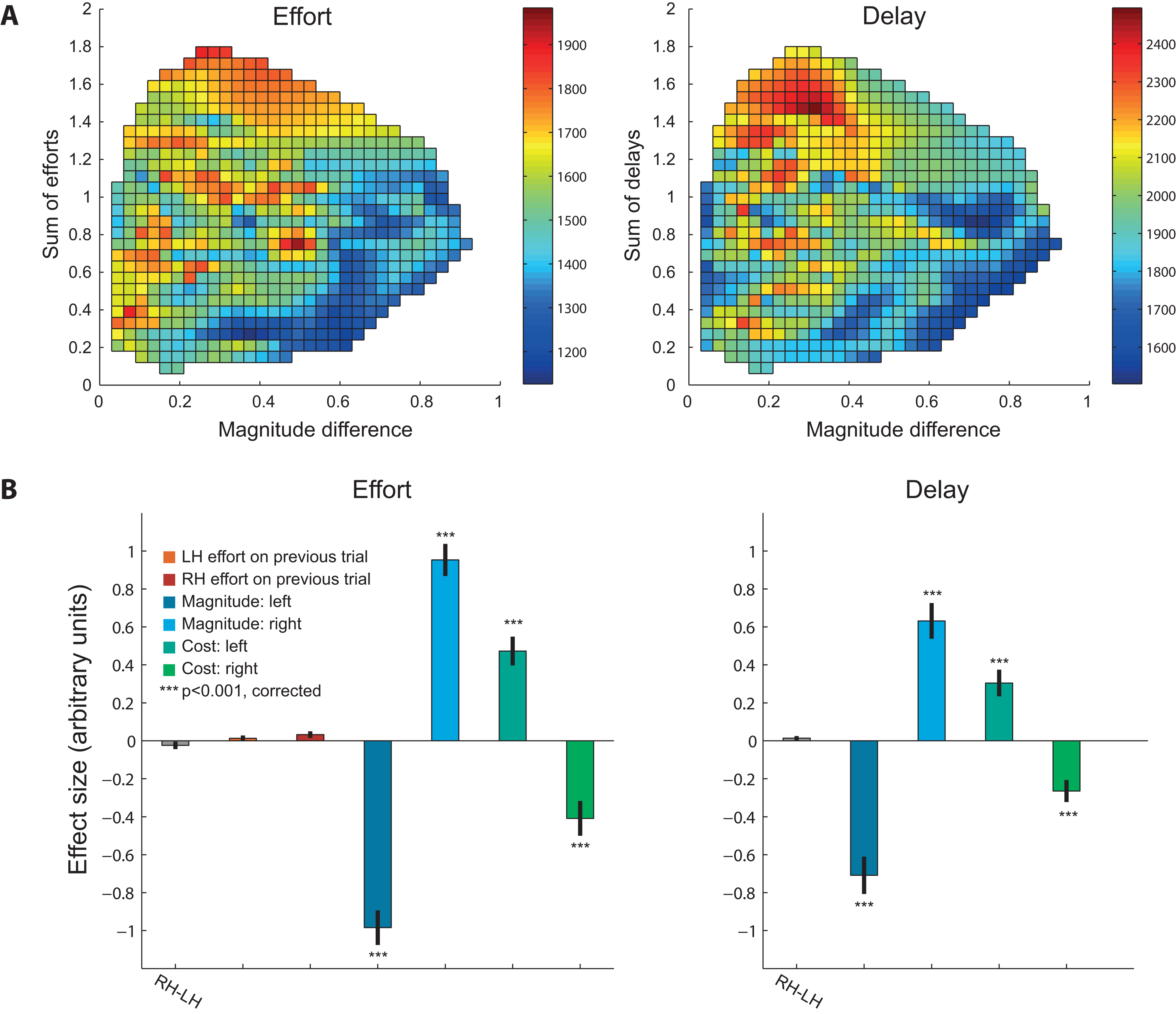

Supplement: S1 Fig — A, Response times are shown as a function of the difference in reward magnitude between the two choice options, and the sum of the costs of the two choice options, separately for effort costs (left) and delay costs (right). While large reward differences speed up the choice process, larger overall costs slow response times. This effect is observed independent of the type of cost. B, Mean (± SEM) parameter estimates from a logistic regression analysis of each participant’s choice pattern. Participants’ choices were driven by both options’ reward magnitude and cost level, showing that all dimensions of the outcomes were taken into consideration for computing a choice. Benefits and costs had opposite effects: larger costs discouraged and larger reward magnitudes encouraged the choice of an option. (TIF) [file pcbi.1004116.s002.tif]

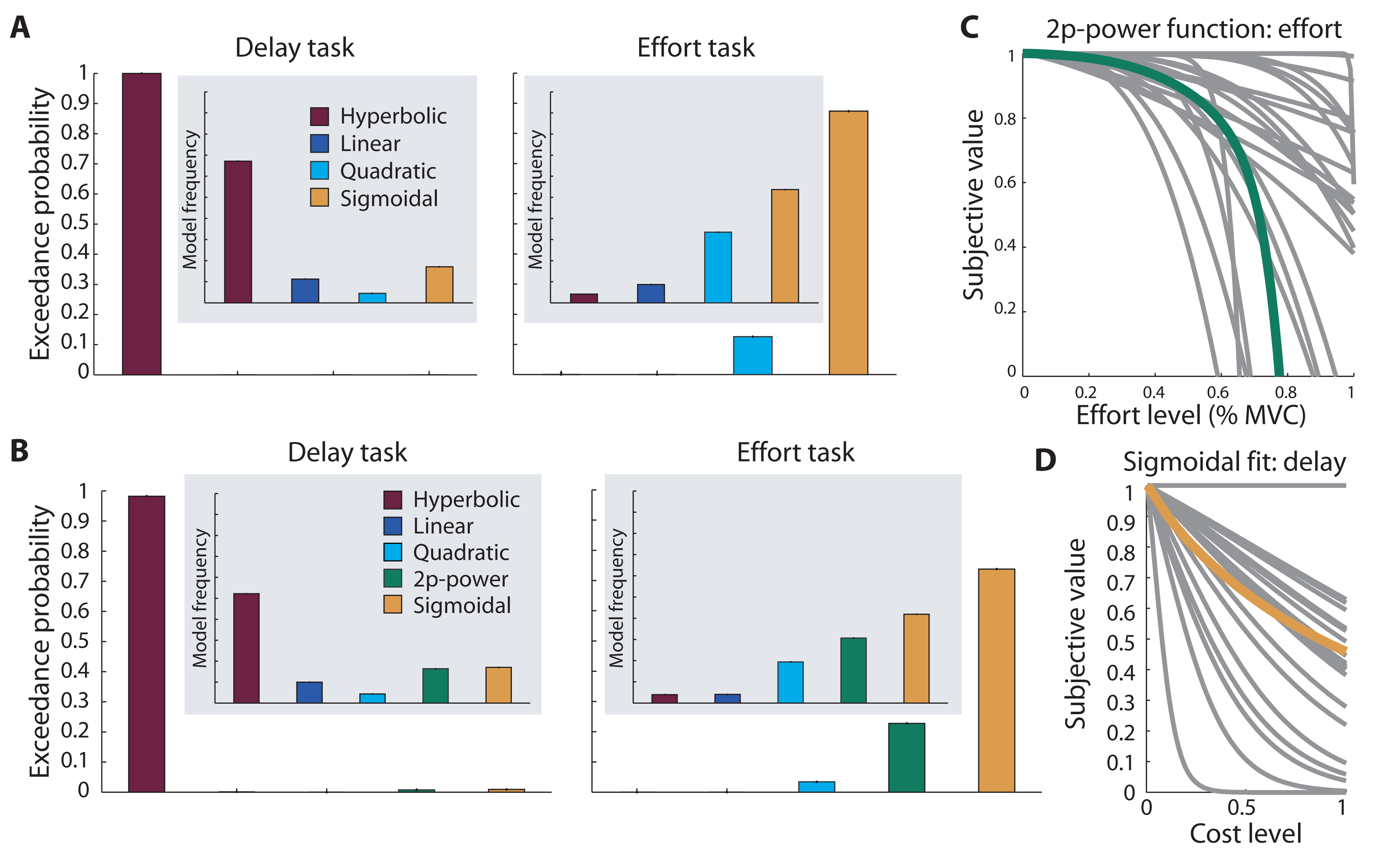

Supplement: S2 Fig — A-B, Bayesian Model Comparison comparing all four (A) or five (B) behavioral models: in both comparisons, the hyperbolic model provides the best explanation for choices on the delay task. In the effort task, the other concave models (quadratic in A and quadratic + two-parameter power function in B) provide an almost comparable explanation of choices as the sigmoidal model. This is because choice stimuli were not individually adjusted and optimized to distinguish similar discounting shapes, but instead designed to distinguish hyperbolic and concave discounting behaviors. C, The individual and average fits obtained from a two-parameter flexible power function show that this model entails a steep decline towards negative values. This model has less evidence than the sigmoidal model despite having the same number of parameters and similar flexibility in the lower range of efforts. D, Sigmoidal fit to delay choices shows that the same model produces an opposite shape in the delay compared to the effort version of the task (compare Fig. 3C). (TIF) [file pcbi.1004116.s003.tif]

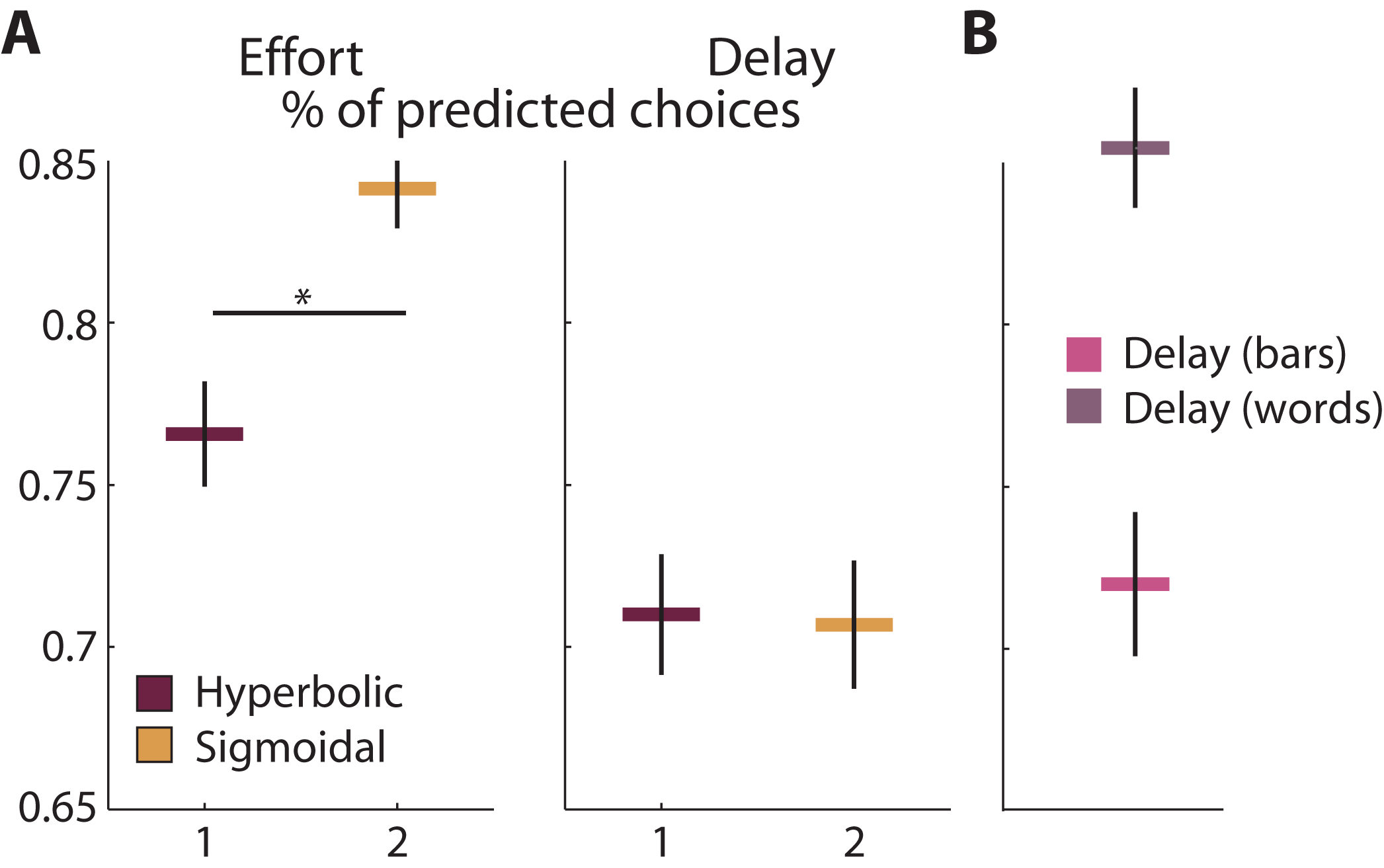

Supplement: S3 Fig — A, Percentage of correctly predicted choices for the hyperbolic and sigmoidal models. Note that the percentage of correctly predicted choices does not take into account the additional model parameter of the sigmoidal model, which importantly was considered in the formal model comparison results shown in Fig. 3B. B, Percentage of correctly predicted choices in a subset of 14 of 23 participants who in addition to the original delay task (with bars signaling the delay, as for effort) also completed a version that indicated delays using words. In the latter task, we were able to predict a much higher percentage of choices. All error bars/vertical bars denote SEM. (TIF) [file pcbi.1004116.s004.tif]

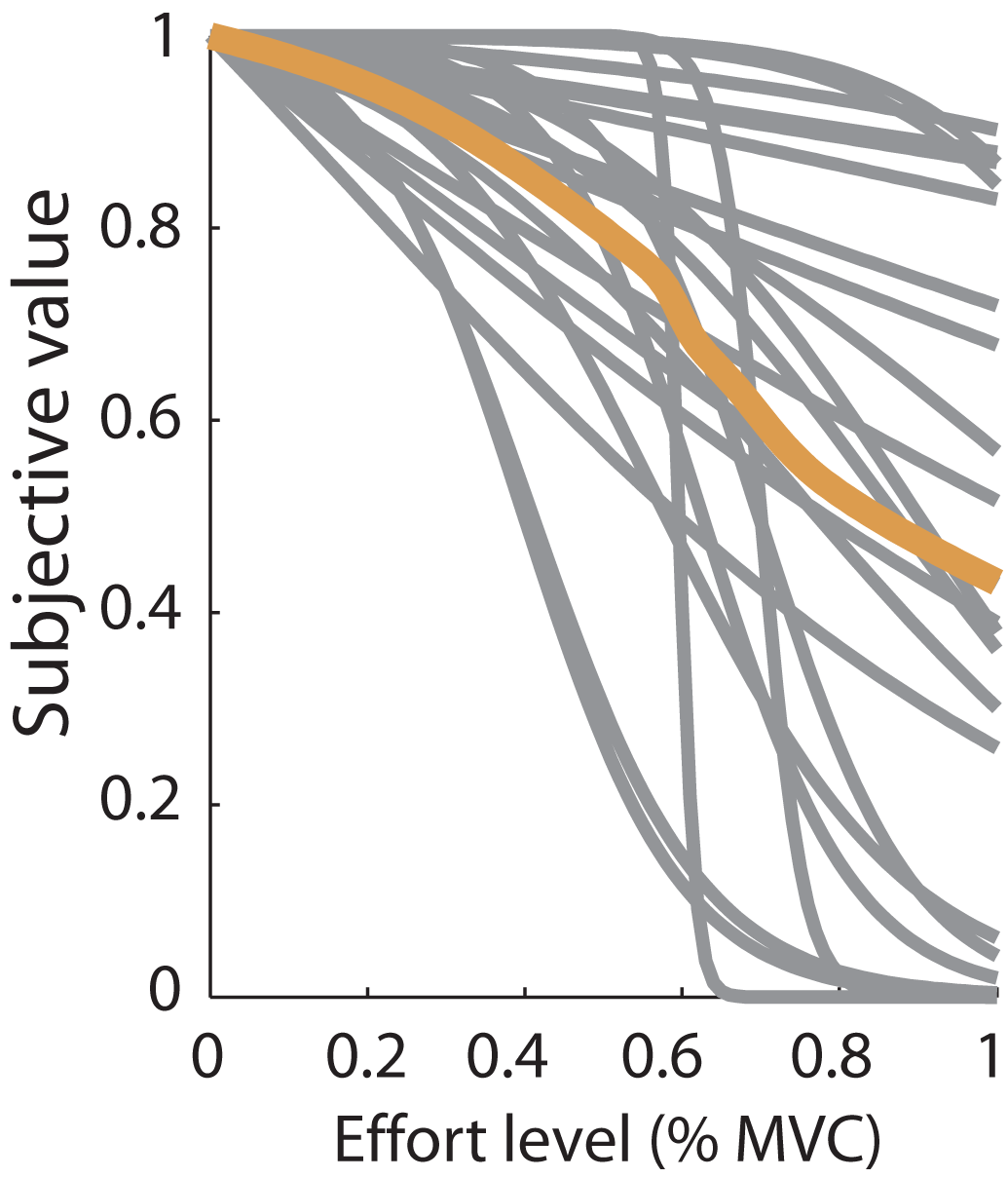

Supplement: S4 Fig — The individual and group fits of the winning sigmoidal model for Experiment 1 are shown as in Fig. 3C (left), but here, instead of using the required force, analyses were based on the force level actually produced in a given trial (i.e., %MVC here refers to the produced effort level). However, because not all trials involved an effort production, the ‘produced’ effort had to be predicted for all trials (predicted produced). This was achieved by fitting the force from all effort production trials (see Fig. 2G-H) using a quadratic trend, to predict the force given the trial’s required force level. Overall, the ‘predicted produced’ force slightly exceeded the required force and increased somewhat supra-linearly for higher efforts (see Fig. 2, G-H). However, and importantly, the resulting sigmoidal fits from this analysis were qualitatively similar to the original analysis, suggesting that using the predicted produced force does not change any of the conclusions. (TIF) [file pcbi.1004116.s005.tif]

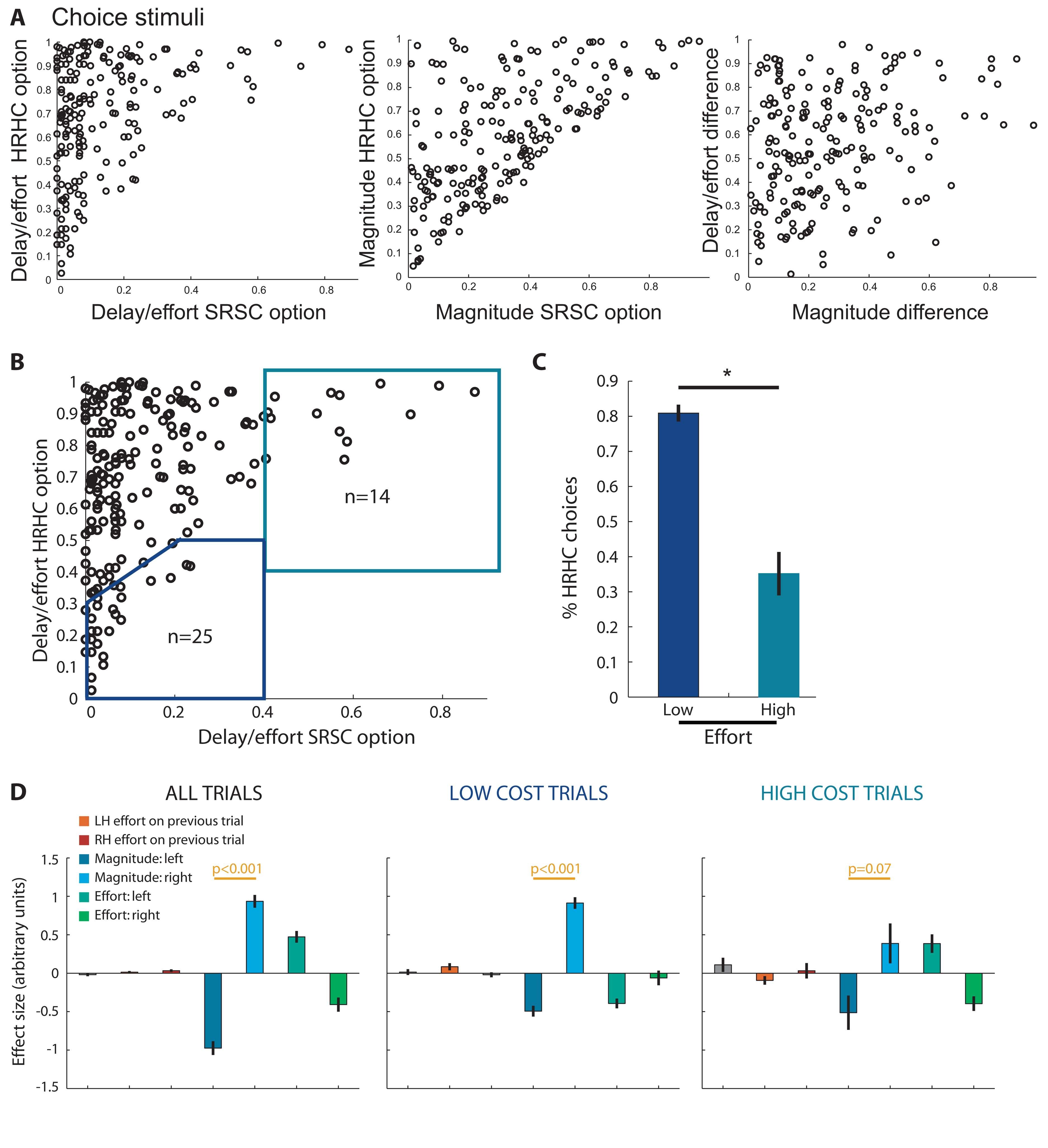

Supplement: S5 Fig — A, The choice stimuli were carefully designed to cover the entire range of magnitudes and costs, and magnitude and cost differences. They were also optimized for distinguishing between concave and convex behavioral models. B, To illustrate that the percentage of choices explained by a model depends on the offered choice stimuli (see Results), we repeated the model fitting and logistic regression analysis on the highlighted subset of trials which was defined as choices in which (a) both options were in the lower cost range, with at least one option with cost <0.2, and the difference to the second option not higher than 0.3, or (b) both options were in the higher range of cost levels (both costs>0.4). This included 25 choices for the lower and 14 choices for the higher cost range. C, The percentage of choices of the higher-reward/higher-cost (HRHC) option was higher in the subset of trials with small efforts compared to large efforts. D, Furthermore, reward magnitude (blue bars) influenced choices less on these high cost (right), compared to low cost (center) trials. (TIF) [file pcbi.1004116.s006.tif]
