# Supplementary material for: Behavioral Modeling of Human Choices Reveals Dissociable Effects of Physical Effort and Temporal Delay on Reward Devaluation
Source: PLoS Comput Biol. 2015 Mar 27;11(3):e1004116. doi: 10.1371/journal.pcbi.1004116 (PMC4376637; doi:10.1371/journal.pcbi.1004116)
Supplement: S1 Table — For each subject in Experiment 1, the table shows the parameter estimates for the hyperbolic (k) and sigmoidal model (k and p; see fits in Fig. 3C). (DOCX) [file pcbi.1004116.s007.docx]

**S1 Table**

| **Model parameters: Experiment 1** | | | | | | | |
| --- | --- | --- | --- | --- | --- | --- | --- |
|  | *Effort* | | | | *Delay* | |  |
|  | hyperbolic | sigmoidal | | | hyperbolic | sigmoidal | |
|  | k | k | *p* | | k | k | *p* |
| **s1** | 5.92 | 96.02 | | 0.54 | 1.27 | 1.27 | 0.03 |
| **s2** | 0.96 | 5.23 | | 0.88 | 0.92 | 1.23 | 0.24 |
| **s3** | 1.49 | 3.72 | | 0.70 | 0.01 | 9.95 | 1.98 |
| **s4** | 5.34 | 41.77 | | 0.61 | 0.62 | 1.04 | 0.49 |
| **s5** | 4.56 | 10.65 | | 0.57 | 2.21 | 1.94 | 0.14 |
| **s6** | 0.94 | 1.25 | | 0.26 | 1.32 | 1.45 | 0.18 |
| **s7** | 0.53 | 0.86 | | 0.46 | 0.86 | 1.12 | 0.22 |
| **s8** | 0.14 | 4.46 | | 1.34 | 0.01 | 10.06 | 2.03 |
| **s9** | 5.59 | 6.79 | | 0.50 | 3.13 | 2.11 | 0.03 |
| **s10** | 0.16 | 0.49 | | 2.13 | 8.13 | 3.97 | 0.01 |
| **s11** | 1.33 | 4.43 | | 0.78 | 13.19 | 3.52 | 0.02 |
| **s12** | 1.33 | 5.72 | | 0.80 | 0.62 | 0.99 | 0.55 |
| **s13** | 3.27 | 11.51 | | 0.64 | 18.18 | 6.33 | 0.08 |
| **s14** | 1.28 | 1.63 | | 0.34 | 1.41 | 1.59 | 0.22 |
| **s15** | 0.35 | 12.08 | | 0.96 | 0.78 | 1.21 | 0.44 |
| **s16** | 236.46 | 9.26 | | 0.32 | 0.01 | 10.03 | 2.01 |
| **s17** | 33.58 | 10.23 | | 0.33 | 1.22 | 1.35 | 0.06 |
| **s18** | 2.40 | 2.08 | | 0.14 | 0.56 | 0.94 | 0.54 |
| **s19** | 0.18 | 0.40 | | 1.06 | 1.34 | 1.56 | 0.26 |
| **s20** | 1.65 | 1.68 | | 0.13 | 5.64 | 3.18 | 0.12 |
| **s21** | 0.34 | 9.76 | | 0.98 | 1.69 | 1.52 | 0.23 |
| **s22** | 0.44 | 0.78 | | 0.62 | 48.66 | 18.02 | 0.03 |
| **s23** | 0.19 | 0.42 | | 1.23 | 0.01 | 9.97 | 1.99 |
|  |  |  | |  |  |  |  |
| **Mean** | 13.41 | 10.49 | | 0.71 | 4.86 | 4.10 | 0.52 |
| **SEM** | 10.24 | 4.28 | | 0.09 | 2.20 | 0.94 | 0.15 |
|  |  |  | |  |  |  |  |

**S1 Table, Individual parameter estimates (Experiment 1)**

For each subject in Experiment 1, the table shows the parameter estimates for the hyperbolic (k) and sigmoidal model (k and *p*; see fits in **Fig 3C**).
