# Supplementary material for: Behavioral Modeling of Human Choices Reveals Dissociable Effects of Physical Effort and Temporal Delay on Reward Devaluation
Source: PLoS Comput Biol. 2015 Mar 27;11(3):e1004116. doi: 10.1371/journal.pcbi.1004116 (PMC4376637; doi:10.1371/journal.pcbi.1004116)
Supplement: S2 Table — Accuracy, complexity and log-evidence values estimated for each participant and model. These values determine the results of the Bayesian model comparison shown in Fig. 3B (Experiment 1). Note that log model evidence = accuracy—complexity (DOCX) [file pcbi.1004116.s008.docx]

**S2 Table**

|  | **Accuracy and complexity: Experiment 1** | | | | | | | | | | | | | | | | | | | |
| --- | --- | --- | --- | --- | --- | --- | --- | --- | --- | --- | --- | --- | --- | --- | --- | --- | --- | --- | --- | --- |
|  | *Effort* | | | | | | | | | *Delay* | | | | | | | | | |  |
|  | **ACCURACY** | | **COMPLEXITY** | | | | **MODEL EVIDENCE** | | | **ACCURACY** | | **COMPLEXITY** | | | | **MODEL**  **EVIDENCE** | | | |  |
|  | **hyp** | **sig** | **hyp** | | **sig** | | **hyp** | | **sig** | **hyp** | **sig** | **hyp** | | **sig** | | **hyp** | | **sig** | |  |
| **s1** | -172.14 | -36.92 | 5.12 | 12.84 | | -177.26 | | -49.76 | | -97.27 | -109.75 | 6.81 | 7.32 | | -104.08 | | -117.07 | |  |  |
| **s2** | -150.73 | -134.72 | 6.03 | 10.32 | | -156.77 | | -145.04 | | -91.10 | -84.76 | 6.85 | 7.93 | | -97.95 | | -92.70 | |  |  |
| **s3** | -146.98 | -138.17 | 5.89 | 9.53 | | -152.87 | | -147.70 | | -181.06 | -181.15 | 2.77 | 2.71 | | -183.82 | | -183.86 | |  |  |
| **s4** | -180.55 | -30.01 | 4.70 | 11.87 | | -185.25 | | -41.88 | | -178.49 | -178.33 | 4.99 | 5.33 | | -183.47 | | -183.66 | |  |  |
| **s5** | -166.65 | -81.46 | 5.46 | 11.63 | | -172.11 | | -93.09 | | -173.83 | -174.10 | 5.58 | 6.27 | | -179.41 | | -180.37 | |  |  |
| **s6** | -107.67 | -101.30 | 6.63 | 7.58 | | -114.30 | | -108.88 | | -164.61 | -163.99 | 5.97 | 6.57 | | -170.58 | | -170.55 | |  |  |
| **s7** | -109.73 | -107.36 | 6.35 | 7.04 | | -116.08 | | -114.40 | | -156.07 | -156.79 | 5.95 | 6.34 | | -162.02 | | -163.13 | |  |  |
| **s8** | 1.91 | 13.26 | 6.05 | 10.99 | | -4.14 | | 2.27 | | -182.19 | -182.22 | 2.68 | 2.67 | | -184.87 | | -184.89 | |  |  |
| **s9** | -136.93 | -76.59 | 5.79 | 10.91 | | -142.72 | | -87.50 | | -71.41 | -70.65 | 6.81 | 8.07 | | -78.22 | | -78.72 | |  |  |
| **s10** | -25.71 | -25.57 | 5.94 | 6.24 | | -31.64 | | -31.81 | | -62.52 | -101.49 | 6.57 | 7.44 | | -69.09 | | -108.92 | |  |  |
| **s11** | -124.53 | -80.40 | 6.56 | 11.31 | | -131.09 | | -91.71 | | -62.03 | -68.23 | 6.32 | 8.04 | | -68.35 | | -76.27 | |  |  |
| **s12** | -147.71 | -110.27 | 6.22 | 11.09 | | -153.93 | | -121.36 | | -177.18 | -177.10 | 4.94 | 5.20 | | -182.12 | | -182.30 | |  |  |
| **s13** | -170.95 | -121.13 | 5.46 | 10.55 | | -176.40 | | -131.68 | | -179.40 | -177.35 | 4.30 | 5.19 | | -183.71 | | -182.54 | |  |  |
| **s14** | -148.12 | -143.15 | 6.18 | 7.75 | | -154.31 | | -150.90 | | -178.34 | -177.74 | 5.26 | 5.95 | | -183.61 | | -183.69 | |  |  |
| **s15** | -94.54 | -62.22 | 6.21 | 12.51 | | -100.75 | | -74.73 | | -177.22 | -176.50 | 5.30 | 5.81 | | -182.52 | | -182.31 | |  |  |
| **s16** | -140.11 | -54.10 | 4.59 | 11.84 | | -144.70 | | -65.94 | | -184.17 | -184.18 | 2.59 | 2.59 | | -186.76 | | -186.77 | |  |  |
| **s17** | -131.71 | -5.33 | 5.10 | 14.87 | | -136.81 | | -20.20 | | -82.16 | -82.33 | 6.96 | 7.78 | | -89.12 | | -90.12 | |  |  |
| **s18** | -136.57 | -128.69 | 6.29 | 7.50 | | -142.86 | | -136.18 | | -173.40 | -173.41 | 5.15 | 5.42 | | -178.55 | | -178.83 | |  |  |
| **s19** | -145.80 | -145.64 | 4.67 | 4.66 | | -150.46 | | -150.30 | | -175.75 | -174.56 | 5.44 | 6.23 | | -181.19 | | -180.79 | |  |  |
| **s20** | -146.33 | -142.89 | 6.28 | 7.21 | | -152.62 | | -150.10 | | -179.73 | -179.37 | 4.83 | 5.68 | | -184.56 | | -185.05 | |  |  |
| **s21** | -120.18 | -88.86 | 5.66 | 11.61 | | -125.84 | | -100.46 | | -188.26 | -188.67 | 4.41 | 4.77 | | -192.67 | | -193.44 | |  |  |
| **s22** | -76.17 | -74.13 | 6.48 | 7.18 | | -82.64 | | -81.32 | | -180.66 | -185.05 | 3.90 | 6.02 | | -184.55 | | -191.08 | |  |  |
| **s23** | -92.23 | -92.34 | 5.92 | 5.95 | | -98.15 | | -98.29 | | -184.22 | -184.28 | 2.59 | 2.57 | | -186.81 | | -186.85 | |  |  |
|  |  |  |  |  | |  | |  | |  |  |  |  | |  | |  | |  |  |
| **Mean** | -124.79 | -85.56 | 5.81 | 9.70 | | -130.60 | | -95.21 | | -151.35 | -153.57 | 5.09 | 5.73 | | -156.44 | | -159.19 | |  |  |
| **SEM** | 9.29 | 9.68 | 0.13 | 0.55 | | 9.24 | | 9.35 | | 9.53 | 8.80 | 0.30 | 0.36 | | 9.32 | | 8.51 | |  |  |

**S2 Table, Accuracy, complexity, and log-evidence (Experiment 1)**

Accuracy, complexity and log-evidence values estimated for each participant and model. These values determine the results of the Bayesian model comparison shown in **Fig 3B** (Experiment 1). Note that log model evidence = accuracy – complexity.
