# Supplementary material for: Behavioral Modeling of Human Choices Reveals Dissociable Effects of Physical Effort and Temporal Delay on Reward Devaluation
Source: PLoS Comput Biol. 2015 Mar 27;11(3):e1004116. doi: 10.1371/journal.pcbi.1004116 (PMC4376637; doi:10.1371/journal.pcbi.1004116)
Supplement: S3 Table — Our main analysis reported in the manuscript was based on the required force level. The table shows the result of a control analysis which used the produced (‘predicted produced’) rather than required force level for Bayesian parameter estimation and model comparison (mp = mean posterior; xp = exceedance probability, values refer to models 1–2 in Experiment 1). The produced force was predicted from a quadratic model fitted to each participant’s force produced in effort production trials. None of the conclusions change with this analysis, showing that the concave shape of effort discounting is not due to slight increases in the produced force and its variability observed with increasing effort levels. (DOCX) [file pcbi.1004116.s009.docx]

**S3 Table**

| **Experiment 1** | **mp** | **xp** |
| --- | --- | --- |
| Required force (original analysis) | 0.04 0.96 | 0.00 1.00 |
| Produced force (control analysis) | 0.05 0.95 | 0.00 1.00 |

**S3 Table, Exerted versus required force (Experiment 1)**

Our main analysis reported in the manuscript was based on the required force level. The table shows the result of a control analysis which used the produced (‘predicted produced’) rather than required force level for Bayesian parameter estimation and model comparison (mp = mean posterior; xp = exceedance probability, values refer to models 1-2 in Experiment 1). The produced force was predicted from a quadratic model fitted to each participant’s force produced in effort production trials. None of the conclusions change with this analysis, showing that the concave shape of effort discounting is not due to slight increases in the produced force and its variability observed with increasing effort levels.
