# Supplementary material for: Behavioral Modeling of Human Choices Reveals Dissociable Effects of Physical Effort and Temporal Delay on Reward Devaluation
Source: PLoS Comput Biol. 2015 Mar 27;11(3):e1004116. doi: 10.1371/journal.pcbi.1004116 (PMC4376637; doi:10.1371/journal.pcbi.1004116)
Supplement: S4 Table — Experiment 1. Left: The percentage of higher-reward/higher-cost choices on trials following force-production trials, compared to an equally large subset of trials following a no-effort trial. Right: Percentage of HRHC choices on trials following an easy compared to a hard realized effort (median split). The absence of statistical differences shows that choice behavior was not affected by local trial-by-trial fatigue. (DOCX) [file pcbi.1004116.s010.docx]

**S4 Table**

| **% choices of HRHC option: analysis of trial-by-trial fatigue** | | | | |
| --- | --- | --- | --- | --- |
|  | **Post-effort** | **Post-no-effort** | **Post-easy-effort** | **Post-hard-effort** |
| **s1** | 0.37 | 0.28 | 0.38 | 0.24 |
| **s2** | 0.64 | 0.58 | 0.41 | 0.33 |
| **s3** | 0.58 | 0.50 | 0.59 | 0.85 |
| **s4** | 0.37 | 0.34 | 0.24 | 0.22 |
| **s5** | 0.39 | 0.35 | 0.23 | 0.24 |
| **s6** | 0.59 | 0.59 | 0.47 | 0.46 |
| **s7** | 0.69 | 0.75 | 0.61 | 0.74 |
| **s8** | 0.93 | 0.94 | 0.92 | 0.96 |
| **s9** | 0.34 | 0.28 | 0.18 | 0.20 |
| **s10** | 0.90 | 0.92 | 0.77 | 0.80 |
| **s11** | 0.54 | 0.58 | 0.46 | 0.50 |
| **s12** | 0.58 | 0.62 | 0.48 | 0.50 |
| **s13** | 0.38 | 0.41 | 0.24 | 0.06 |
| **s14** | 0.47 | 0.59 | 0.45 | 0.41 |
| **s15** | 0.80 | 0.85 | 0.50 | 0.82 |
| **s16** | 0.20 | 0.12 | 0.21 | 0.33 |
| **s17** | 0.25 | 0.15 | 0.20 | 0.13 |
| **s18** | 0.41 | 0.45 | 0.40 | 0.46 |
| **s19** | 0.71 | 0.76 | 0.83 | 0.58 |
| **s20** | 0.48 | 0.54 | 0.39 | 0.41 |
| **s21** | 0.78 | 0.79 | 0.78 | 0.73 |
| **s22** | 0.73 | 0.80 | 0.75 | 0.64 |
| **s23** | 0.86 | 0.87 | 0.79 | 0.70 |
|  |  |  |  |  |
| **Mean** | 0.56 | 0.56 | 0.49 | 0.49 |
| **SEM** | 0.04 | 0.05 | 0.05 | 0.05 |

**S4 Table, Percentage of high-cost choices (Experiment 1)**

**Experiment 1, Left:** The percentage of higher-reward/higher-cost choices on trials following force-production trials, compared to an equally large subset of trials following a no-effort trial. **Right:** Percentage of HRHC choices on trials following an easy compared to a hard realized effort (median split). The absence of statistical differences shows that choice behavior was not affected by local trial-by-trial fatigue.
